# Biophysical and Pharmacological Characterization of Energy-Dependent Efflux of Sb in Laboratory-Selected Resistant Strains of *Leishmania* (*Viannia*) Subgenus

**DOI:** 10.3389/fcell.2017.00024

**Published:** 2017-03-24

**Authors:** Priscila G. dos Reis, Rubens L. do Monte-Neto, Maria N. Melo, Frédéric Frézard

**Affiliations:** ^1^Departamento de Fisiologia e Biofísica, Instituto de Ciências Biológicas, Universidade Federal de Minas GeraisBelo Horizonte, Brazil; ^2^Departamento de Farmácia/Ensino e Pesquisa, Hospital João XXIII - Fundação Hospitalar do Estado de Minas GeraisBelo Horizonte, Brazil; ^3^Laboratório de Parasitologia Celular e Molecular, Centro de Pesquisas René Rachou – CPqRR/FIOCRUZBelo Horizonte, Brazil; ^4^Departamento de Parasitologia, Instituto de Ciências Biológicas, Universidade Federal de Minas GeraisBelo Horizonte, Brazil

**Keywords:** *Leishmania*, *Viannia*, antimony, resistance, chemosensitizer, ABC transporter, efflux, influx

## Abstract

The growing resistance of leishmaniasis to first-line drugs like antimonials in some regions limits the control of this parasitic disease. The precise mechanisms involved in *Leishmania* antimony resistance are still subject to debate. The reduction of intracellular Sb^III^ accumulation is a common change observed in both laboratory-selected and field isolated resistant *Leishmania* strains, but the exact transport pathways involved in antimony resistance have not yet been elucidated. In order to functionally characterize the antimony transport routes responsible for resistance, we performed systematic transport studies of Sb^III^ in wild-type and resistant strains of *L*. (*Viannia*) *guyanensis* and *L*. (*V.) braziliensis*. Those include influx and efflux assays and the influence of ABC transporters and metabolism inhibitors: prochlorperazine, probenecid, verapamil, BSO, and sodium azide. The mRNA levels of genes associated with antimony resistance (*MRPA, GSH1, ODC, AQP1, ABCI4*, and *ARM58*) were also investigated in addition to intracellular thiol levels. A strong reduction of Sb influx was observed in *L. guyanensis* resistant mutant (LgSbR), but not in *L. braziliensis* (LbSbR). Both mutants showed increased energy-dependent efflux of Sb^III^, when compared to their respective parental strains. In LgSbR, BSO and prochlorperazine inhibited antimony efflux and resistance was associated with increased *MRPA* and *GSH1* mRNA levels, while in LbSbR antimony efflux was inhibited by probenicid and prochlorperazine in absence of resistance-associated gene modulation. Intracellular thiol levels were increased in both Sb-resistant mutants. An energy-dependent Sb^III^ efflux pathway sensitive to prochlorperazine was clearly evidenced in both Sb-resistant mutants. In conclusion, the present study allowed the biophysical and pharmacological characterization of energy-dependent Sb efflux pathway apparently independent of MRPA, ABCI4, and ARM58 upregulation, in *Leishmania* (Vianna) mutant selected *in vitro* for resistance to Sb^III^. Prochlorperazine has also been identified as an effective chemosensitizer in both Sb resistant mutants, which acts through inhibition of the active efflux of Sb.

## Introduction

Protozoan parasites belonging to *Leishmania* genus are the causative agents of leishmaniasis that produces a wide spectrum of clinical disease in humans ranging from self-healing cutaneous (CL) and mucocutaneous (MCL) lesions to fatal visceral (VL) infection, if not treated (Murray et al., 2005). The disease is a public health concern, endemic in 98 countries reaching up to 1.2 million new cases annually and affecting mainly poor and marginalized populations (Alvar et al., 2012). In the New World, *Leishmania* (*Viannia*) *braziliensis* and *Leishmania* (*Viannia*) *guyanensis* cause cutaneous and mucocutaneous leishmaniasis (MCL) form of the disease (Marzochi and Marzochi, 1994; Murray et al., 2005).

The pentavalent antimony (Sb^V^) derivatives, sodium stibogluconate (Pentostam® and meglumine antimoniate (Glucantime®), have been used in the treatment of the majority of cases of leishmaniasis for almost 70 years worldwide. Those are considered as prodrugs that are activated through reduction of Sb^V^ to Sb^III^ (Frézard et al., 2009). Currently, these drugs have two main limitations. First, side effects are frequent and can be fatal. Second, parasite resistance is emerging in some endemic areas, causing an increase in treatment failure (Lira et al., 1999; Hadighi et al., 2006) with major incidence in India, where 65% of patients are refractories to treatment (Perry et al., 2011).

Studies concerning experimental resistance to antimony in *Leishmania* indicate that several mechanisms may occur, even concomitantly in the same parasite (Ouellette et al., 2004; Decuypere et al., 2005, 2012; Croft et al., 2006; Mukherjee et al., 2007; Do Monte-Neto et al., 2011; Kumar et al., 2012; Berg et al., 2013; Kazemi-Rad et al., 2013; Cheng and Sun, 2014). The resistance to Sb in *Leishmania* usually involves a reduction in the intracellular drug accumulation (Callahan et al., 1994; Dey et al., 1994; Brochu et al., 2003). The upregulation of the ABC transporter multidrug resistance-associated protein A (MRPA), identified in intracellular vesicular membranes, is a common change observed in both field isolates and laboratory-selected *Leishmania* resistant strains (Papadopoulou et al., 1994; Legaré et al., 2001; Decuypere et al., 2005; Mukherjee et al., 2007; Moreira et al., 2013). In some resistant mutants, including the *L. guyanensis* strain studied here, Sb^III^ entry was found to be reduced through either down regulation (Marquis et al., 2005), deletion or a point mutation (Monte-Neto et al., 2015) of the aquaglyceroporin 1 (AQP1) gene. In a recent review, Frézard et al. (2014) pointed out that attempts to characterize the transport pathways of Sb^III^ in resistant strains overexpressing the MRPA transporter showed apparently conflicting results, with either increased efflux (Dey et al., 1994) or decreased influx (Callahan et al., 1994) and that other means of transport, besides the sequestration of Sb in intracellular vesicles, may contribute to the resistance of *Leishmania* to Sb, such as the efflux of Sb^III^ by a transporter yet to be identified. Recently, three different membrane proteins were proposed for their putative involvement in Sb^III^ efflux in resistant *Leishmania* parasites. Manzano et al. (2013) and Perea et al. (2016) identified two distinct ABC transporters in *L. major* capable of promoting Sb^III^ and thiol efflux, thereby conferring resistance to antimonials. One of these transporters is a member of ABCI subfamily (LABCI4) and the other one is the ABC protein LABCG2. Both transporters were found to be partially located in the plasma membrane and it was hypothesized that they may confer Sb resistance by sequestering metal-thiol conjugates within vesicles and through further exocytosis by means of the parasite's flagellar pocket. Another membrane protein called ARM58 (antimony resistance marker of 58 kDa), when overexpressed in *L. braziliensis* (Nühs et al., 2013) and *L. infantum* (Schäfer et al., 2014), also promoted resistance to Sb through reduced drug accumulation and presumably increased efflux of thiol-Sb conjugate. Interestingly, ARM58 was found to be localized near the flagellar pocket hints but, contrary to LABCG2 and LABCl4, it did not seem to mediate energy-dependent transport activity. Indeed, ARM58 is part of a subtelomeric cluster comprising the neighboring genes ARM56 and HSP23, which confers antimony resistance by inducing exosome-mediated secretion (Tejera Nevado et al., 2016). Using a new approach called Cos-Seq—that combines functional cloning and massive next-generation sequencing, Gazanion et al. (2016) have confirmed the up-regulation of ARM58 in laboratory-selected antimony-resistant *L. infantum* (Gazanion et al., 2016).

Although the mechanisms of *Leishmania* resistance to Sb has been extensively studied from the molecular point of view, systematic functional studies involving biophysical and pharmacological approaches to characterize the precise transport pathways of Sb are scarce. In this context, the present work aimed to characterize the transport routes of Sb in *L. braziliensis* and *L. guyanensis* strains selected for their resistance to Sb^III^ (LbSbR and LgSbR), by using systematic transport kinetic analysis and investigating the effect of ABC transporter inhibitors on the cytotoxicity, uptake and efflux of Sb.

## Materials and methods

### Chemicals

Probenecid, prochlorperazine, verapamil, potassium antimonyl tartrate hydrate, kanamycin, L-buthionine-(SR)-sulfoximine (BSO), sodium azide (NaN_3_), HEPES, biopterin, ampicillin, L-glutamine and hemin were obtained from Sigma-Aldrich (St Louis, USA). Nitric acid (65%) was obtained from Merck Brasil (Rio de Janeiro, RJ, Brazil).

### *Leishmania* strains and Sb^III^ sensitivity assay

Promastigote forms of two different New World *Leishmania* species: *Leishmania* (*Viannia*) *guyanensis* (MHOM/BR/1975/M4147) and *Leishmania* (*Viannia*) *braziliensis* (MHOM/BR/1975/M2904) were used. Parasites of both strains were selected *in vitro* for resistance to Sb^III^ as previously described (Roberts and Rainey, 1993; Moreira et al., 2013). Promastigotes were exposed to increasing Sb^III^ concentrations up to 650 μM (*L. guyanensis*) and 330 μM (*L. braziliensis*) in 25 cm^2^ flasks containing 5 mL of minimum essential culture medium (α-MEM) (Gibco, Invitrogen, NY, USA). The selected parasite strains (*L. braziliensis* Sb^III^330.2 and *L. guyanensis* Sb^III^650.4) were maintained in α-MEM, supplemented with 10% (v/v) heat-inactivated fetal calf serum (Cultilab, Brasil), 100 μg/mL kanamycin, 50 μg/mL ampicillin, 2 mM L-glutamine, 5 μg/mL hemin, 5 μM biopterin, pH 7.0 and incubated at 25°C in a B.O.D. incubator. As previously described, the resistant mutant *L. guyanensis* Sb^III^650.4 harbors a single nucleotide polymorphism at AQP1 coding gene that leads to the point mutation G133D at protein level. Functional analysis revealed that this mutation was directly associated with the reduced antimony uptake (Monte-Neto et al., 2015). On the other hand, *L. braziliensis* resistant mutant presented intact copies of AQP1 as revealed by gene sequencing (Supplementary Figure 1).

To compare the Sb^III^ sensitivity of different strains, mid-log phase wild-type and resistant *Leishmania* promastigotes were inoculated at 10^6^ cells/mL in α-MEM medium in the presence of Sb^III^ (as potassium antimonyl tartrate). Biological replicates in the absence of drug were established as control. The cultures were incubated under shaking at 25 ± 1°C for 72 h and the growth inhibition was determined by measuring the absorbance at 600 nm using a microplate reader (Organon Teknica Microwell), as previously described (Fumarola et al., 2004). Three independent experiments were carried out. The half-maximal inhibitory concentration (IC_50_) values were calculated based on concentration-response curves applying a sigmoidal dose-response equation with variable slope carried out using the software GraphPad Prism version 6.0 (GraphPadSoftware Inc., San Diego, CA, USA).

### Real time qRT-PCR

Total RNA was extracted from 10^8^ mid-log phase *Leishmania* spp. promastigotes using RNeasy Plus mini kit (Qiagen Sciences, Maryland, USA) as described by the manufacturer. First-strand cDNA was synthesized from 2.5 μg of total RNA using Oligo dT12–18 and SuperScript II RNase H-Reverse Transcriptase (Invitrogen, Carlsbad, CA, USA) according to the manufacturer protocol. Equal amounts of cDNA were run in triplicate and amplified in 25 μL reactions containing 1 x iQ SYBR® Green Supermix (Bio-Rad, Hercules, CA, USA), 100 nM forward and reverse primers and 100 ng of cDNA target. Reactions were carried out using a rotator thermocycler Rotor Gene (RG 3000, Corbett Research, San Francisco, USA). Mixtures were initially incubated at 95°C for 5 min and then cycled 30 times at 95°, 60°, and 72°C for 15 s. No-template controls were used as recommended. Three technical and biological replicates were established for each reaction. The relative amount of PCR products generated from each primer set was determined based on the cycle threshold (Ct) value and the amplification efficiencies. Data were analyzed using the comparative 2^−ΔΔCt^ method. Gene expression levels were normalized to constitutively expressed mRNA encoding glyceraldehyde-3-phosphate dehydrogenase (*GAPDH, LbrM.30.2950*). The primers for targeted genes: *MRPA* (*LbrM.23.0280*), *GSH1*—that encodes to gamma-glutamylcysteine synthetase (γGCS-*LbrM.14.0880*), *ODC* (*LbrM.12.0300*), *AQP1* (*LbrM.31.0020*), *ABCI4* (*LbrM.33.3540*), *ARM58* (*LbrM.20.0210*), and internal gene expression control GAPDH were designed using PrimerQuest® (https://www.idtdna.com/Primerquest/Home/Index). Primer sequences are listed in Table 1.

**Table 1:** **Chosen target genes and their primer pairs used for RT-qPCR**.

**Gene product (ID)**	**Sequence of forward and reverse primers**	**Product size (bp)**
***MRPA***		
*LbrM.23.0280*	5′TGTCCACCTGGCCAATGTAGTCTT3′	125
	5′TCGGAAAGACAACCTCCGGCTTTA3′	
***GSH1***		
*LbrM.14.0880*	5′GAACACGGCTGATCAGTACAA3′	118
	5′AAGGTTAGCGTGCTCAAGTC3′	
***ODC***		
*LbrM.12.0300*	5′GTACATCGAGAAGGGTGTGAAG3′	127
	5′GCCGAGGTCAATGATGTAGAA3′	
***AQP1***		
*LbrM.31.0020*	5′TCTCGCCATCAACGATAACC3′	126
	5′CGTGTAGGGTTGAGAGCATATC3′	
***ABCI4***		
*LbrM.33.3540*	5′CTGTAGACGAAGCGGGTATTT3′	135
	5′CTAGGCGATGAGACACCATAAC3′	
***ARM58***		
*LbrM.20.0210*	5′CCCAAGGGCTTTCACCTAAA3′	103
	5′AGCGGTAGATCTTGTCGTATTG3′	

### Total intracellular thiol measurement

Total intracellular thiols were derivatized from deproteinized cell extracts and separated by high-performance liquid chromatography (HPLC) as previously described (Fairlamb et al., 1987; Mukhopadhyay et al., 1996). Briefly, 10 mL of Sb-free logarithmic phase *Leishmania* promastigote cultures were haversted at 0.3–0.4 absorbance (600 nm); washed twice in HEPES/NaCl (21 mM HEPES; 137 mM NaCl; 5 mM KCl; 0.7 mM Na_2_ HPO_4_; 6 mM Glucose; pH 7) and resuspended in HEPES/EDTA (50 mM HEPES; 5 mM EDTA; 1 mM DTT pH 8). At this step, 10 μL were separated for protein dosage by bradford method (Bradford, 1976). Protected from light, 100 μL of 2 mM monobromobimane (mBBr) (Invitrogen, Carlsbad, CA, USA) were added to the samples, mixed and incubated at 70°C for 3 min. Trichloroacetic acid (Fischer Chemical, Atlanta, GA, USA) (200 μL at 25%) were added to the mixture, and the extract was kept at 80°C for at least, 1 h followed by low temperature (4°C) centrifugation at top speed (microcentrifuge) for 20 min. Supernatants were filtered in 0.45-μm filters (Acrodisc Pall, Life Sciences, East Hills, NY, USA) and thiols separated using the liquid chromatograph Shimadzu SCL 10A. Samples were analyzed using a reverse phase column Vydac C18 eluted in methanol 0–100%/acetic acid (25%) gradient pH 3.5. Standard solutions of mBBr-derivatized cysteine (Cys), glutathione (GSH) and trypanothione (TSH) were previously established as calibration curve. Thiols were indirectly measured by mBBR fluorescence at 360 and 450 nm of excitation and emission, respectively, using a coupled fluorescence detector (Shimadzu RF-10Axl).

### Sb uptake

Before performing the assays, Sb^III^-resistant *Leishmania* spp. lines were maintained for at least two passages in α-MEM medium in the absence of Sb^III^, in order to eliminate the residual drug.

The Sb uptake kinetic and influx assays were based on previously described protocols (Roberts and Rainey, 1993; Moreira et al., 2013). Briefly, mid-log phase wild-type and resistant *Leishmania* promastigotes were washed twice in Hepes/Glucose (HG) buffer (20 mM HEPES, 0.15 M NaCl, 10 mM glucose, pH 7.2) and suspended in this buffer at a density of 10^8^ cells/mL.

In the uptake kinetic assay, cells were incubated in the presence of Sb^III^ at 540 μM. In different time points, 1 mL of the cell suspension was harvested and immediately centrifuged at 3,000 × g for 5 min at 4°C and the pellet was washed twice with HG buffer under the same conditions. The pellet was then resuspended in 100 μL HG buffer. A 10-μL aliquot of each sample was used for parasite quantification and viability evaluation and the remaining volume (90 μL) was submitted to digestion in nitric acid (65%). Cell viability was confirmed from the promastigote motility and trypan blue exclusion assay (Freshney, 1994). More than 90% of promastigotes showed motility, except those exposed to sodium azide and more than 95% of cells were considered viable according to trypan blue exclusion. The Sb concentration was determined by graphite furnace atomic absorption spectroscopy (Perkin Elmer, AAnalyst 600). The signal from a blank (cells without Sb) was used for background subtraction. The analytical method for determination of Sb was validated and showed suitable levels of precision, accuracy and linearity. The quantification limit of the analytical method was 0.021 nmol Sb/108 promastigotes. The amount of cellular Sb at the zero time point was equal to 0.044 ± 0.002 nmol Sb/108 promastigotes and, thus, close to the quantification limit, indicating that the binding of Sb to the cell surface is negligible, in agreement with the high hydrophilicity of potassium antimonyl tartrate.

For influx assay, parasites were exposed to Sb^III^ for 1 h at 25°C, at increasing concentrations (0; 100; 250; 500; 1,000; 1,500; 2,000 μM of Sb^III^), in quadruplicates/point. Samples were submitted to the washing procedure mentioned above for removing extracellular Sb traces. Each influx assay was performed three times. The influx rate was calculated as follows: Vi = amount of intracellular antimony/(number of cells x time of uptake). A shorter incubation time could not be used, because of the method quantification limit and loss of accuracy in the determination of the initial rate. Thus, the values determined for initial rate were approximation of the influx rates, especially in the case of WT cells in which Sb uptake was not linear over 1-h time. The Vi-vs-Ce curves were analyzed using the GraphPad Prism 6.0 software to assess whether they best fit with a linear model or the Michaelis-Menten equation as follows:

$$\begin{array}{l} \text{Vi}={\text{Vi}}_{\text{max}}\;\times \;\text{Ce}/\left(\text{Ce}+{\text{K}}_{\text{m}}\right) \end{array}$$

where V_max_ is the maximum influx rate, K_m_ is the Michaelis-Menten constant and Ce is the extracellular concentration of Sb.

The influx rate constant (k_influx_) was calculated as:

$$\begin{array}{l} {\text{k}}_{\text{influx}}={\text{Vi}}_{\text{max}}/{\text{K}}_{\text{m}} \end{array}$$

When no saturation was observed, k_influx_ was determined by linear regression, assuming that Vi = k_influx_ × Ce.

This influx assay was also used to identify the values of Ce at which the mutant and its parental line exhibited the same intracellular concentration of Sb, to be explored in the efflux protocol.

To evaluate the effect of ABC transporter inhibitors on Sb^III^ uptake, mid-log phase *Leishmania* spp. promastigotes were first exposed for 24 h to each of these compounds at non-toxic concentrations (8 μM verapamil, 4 mM probenecid, 10 μM prochlorperazine, 100 μM BSO for resistant strains and 8 μM verapamil, 4 mM probenecid, 3.5 μM prochlorperazine, 100 μM BSO for parental strains). The cells were then resuspended in HG buffer at 10^8^ cells/mL and further exposed to these compounds for 1 h at 25°C, in the presence of 1 mM of Sb^III^. Samples were washed twice with cold HG buffer to remove external Sb and allow the measurement of intracellular Sb. We observed that more than 90% of promastigotes showed motility at the end of the experiment, suggesting that the inhibitors did not act through depletion of intracellular ATP.

When investigating the impact of energy depletion on the uptake of Sb^III^, cells were resuspended in the HG buffer without glucose, but in the presence of 10 mM sodium azide. Those were then incubated for 1 h at 25°C in the presence of 1 mM of Sb^III^ and were subsequently processed as described above.

### Sb efflux

Before performing this assay, Sb^III^-resistant *Leishmania* lines were maintained for at least two passages in α-MEM medium in the absence of Sb^III^, in order to remove the residual drug. Mid-log phase wild-type and resistant *Leishmania* promastigotes were washed twice with HG buffer and resuspended in it at a density of 10^8^ cells/mL. A 1-mL aliquot containing only parasites (blank) was separated and the remaining cells were incubated at 25°C with the concentration of Sb^III^ pre-established in influx assay so as to obtain the same loading of drug in the resistant strain and its parental cells (*L. guyanensis*, 100 μM for WT and 2,000 μM for the mutant; *L. braziliensis*, 500 μM for WT and 2,000 μM for the mutant). After 1 h incubation, the cells were centrifuged at 3,000 × g for 5 min at 4°C, washed twice and resuspended in HG buffer at the original cell density followed by incubation at 25°C. Aliquots of 1 mL were taken from the parasite suspension at 0, 15, 30, 60, and 120 min. Subsequently, these aliquots and the blanks were treated as described above for the quantification of parasite and intracellular Sb. The signal from blanks was considered as background.

Each efflux assay was performed three times in triplicate. Data were plotted as the percentage of initial intracellular Sb content as a function of time and the half-time of Sb efflux was calculated using mono-exponential decay model. The efflux rate constant k_efflux_ was also obtained from the equation:

$$\begin{array}{l} \text{Ve}={\text{k}}_{\text{efflux}}\;\times \;\text{Ci} \end{array}$$

where Ve is the initial rate of efflux determined from the tangent of the curve and Ci is the intracellular concentration of Sb estimated at time zero using a cell volume of 1.2 × 10^−14^ L (Zilberstein and Dwyer, 1984).

To evaluate the interference of ABC transporter inhibitors on Sb efflux, the cells were initially loaded for 1 h at 25°C with 1 mM of Sb^III^ in α-MEM medium. The cells were washed and resuspended in HG buffer at 10^8^ cells/mL. A 1-mL aliquot was immediately removed and processed to determine the initial intracellular amount of Sb^III^. The remaining cells were exposed to the inhibitors at non-toxic concentrations (8 μM verapamil, 4 mM probenecid, 10 μM prochlorperazine, 100 μM BSO, 10 mM sodium azide in resistant strains and 8 μM verapamil, 4 mM probenecid, 3.5 μM prochlorperazine, 100 μM BSO, 10 mM sodium azide in parental strains) for 2 h at 25°C under agitation. Only when evaluating the effect of azide, that the buffer did not contain glucose. The cells were subsequently processed as described above to determine the amount of Sb per cell. We observed that more than 90% of promastigotes showed motility at the end of the experiments when applying verapamil, probenecid, prochlorperazine or BSO, suggesting that these inhibitors did not act through depletion of intracellular ATP.

### Antileishmanial activity of ABC transporter inhibitors and their role as chemosensitizer in SbR *Leishmania*

First, ABC transporter inhibitors (probenecid, prochlorperazine, verapamil and BSO) were evaluated for their antileishmanial activity against SbR and WT *Leishmania* spp. Mid-log phase promastigotes were inoculated at 10^6^ cells/mL in α-MEM medium in the presence of different concentrations of the inhibitors. Biological replicates in the absence of drug were established as control. The cultures were incubated under shaking at 25 ± 1°C for 72 h and the IC_50_s were determined as described above (section *Leishmania* Strains and Sb^III^ Sensitivity Assay). The ability of each inhibitor to sensitize the cells to Sb^III^ was evaluated by performing growth inhibition assay as described above, in the presence of a fixed non-toxic concentration of the inhibitor (8 μM verapamil, 4 mM probenecid, 10 μM prochlorperazine, 100 μM BSO in resistant strains and 8 μM verapamil, 4 mM probenecid, 3.5 μM prochlorperazine, 100 μM BSO in parental strains). The IC_50_ values of Sb^III^ in presence and absence of inhibitor were compared. All experiments were done at least three times as independent experiments performed in triplicate.

### Statistical analyses

The IC_50_ values were calculated by non-linear regression. Data were analyzed by Student's *t*-test or One-way analysis of variance (ANOVA) followed by Bonferroni's multiple comparison test. A *p* ≤ 0.05 was considered statistically significant. All analyses were carried out using the software GraphPad Prism version 6.0 (GraphPad Software Inc., La Jolla, CA, USA).

## Results

### Sb^III^ sensitivity, mRNA levels of resistance markers and thiol levels

While *L. braziliensis* and *L. guyanensis* parental strains presented IC_50_ of Sb^III^ lower than 100 μM, the resistant mutants suffered little influence of Sb^III^ at concentration as high as 600 μM (Table 2 See also growth inhibition curves in Supplementary Figure 2).

**Table 2:** **Half-maximal growth inhibition concentrations (IC_**50**_) of Sb^**III**^ in Sb-resistant ***L. braziliensis*** and ***L. guyanensis*** promastigotes and their respective parental lines and corresponding resistance index**.

**Species**	**IC_50_ (μM)**		**Resistance index**
	**Wild type**	**Resistant**	
*L. (V.) braziliensis*	86.1 ± 1.1	623.7 ± 44.7	7.2
*L. (V.) guyanensis*	47.4 ± 7.8	1167 ± 1.1	24.6

**Figure 1:**
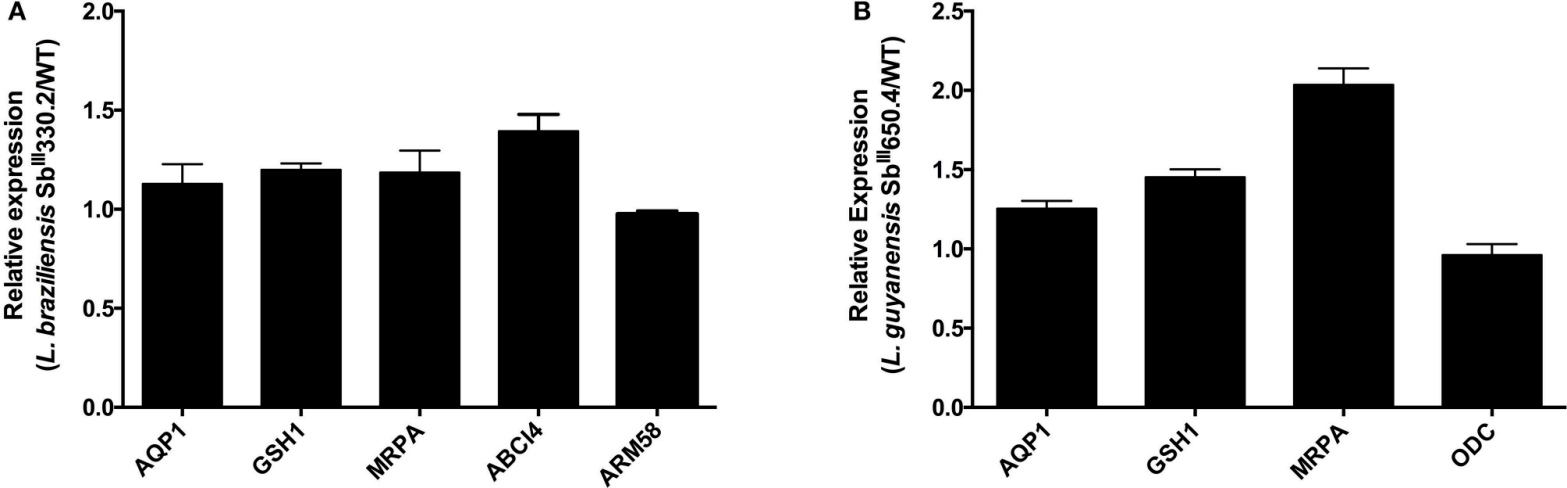
**mRNA levels of classical markers of resistance in Sb-resistant (A)** *L. braziliensis* Sb^III^330.2 and **(B)** *L. guyanensis* Sb^III^650.4 mutants relative to the respective WT parental strains. The mRNA levels were determined by real time PCR. **(A)** *mRNA of, AQP1, GSH1, MRPA, ABCI4*, and *ARM58*. **(B)** mRNA of *AQP1, GSH1, MRPA*, and *ODC*. Results are shown as means of three independent experiments performed from three different RNA preparations.

**Figure 2:**
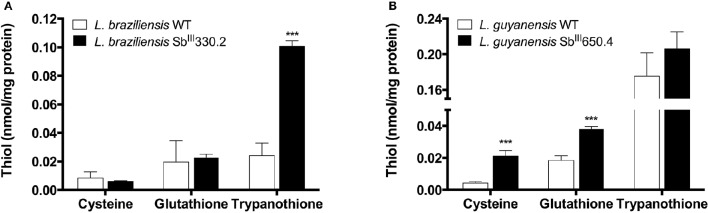
**Intracellular levels of thiols in Sb-resistant ***L. braziliensis*** Sb^**III**^330.2 (A)** and *L. guyanensis* Sb^III^650.4 **(B)** mutants relative to the respective WT parental strains. Thiols were derivatized with monobromobimane from deproteinized cell extract and separated by HPLC coupled with fluorescent detector. The values represent two experiments performed in triplicate. Data were analyzed by one-way ANOVA followed by Dunnet's multiple comparison test. ^***^p < 0.01.

The mRNA levels of *MRPA, GSH1, ODC, AQP1, ABCI4*, and *ARM58*—genes associated with Sb resistance in *Leishmania* parasites—were investigated. As shown in Figure 1B, increased mRNA levels of *MRPA* (2-fold) and to a lower extent of *GSH1* (1.5-fold) were found in LgSbR mutant, when compared to the parental strain. No differences were observed for *ODC* or *AQP1* mRNA for this mutant (Figure 1). On the other hand, LbSbR mutant did not exhibit any relevant change in mRNA levels of either *MRPA, GSH1, AQP1, ABCI4*, or *ARM58* (Figure 1A). Despite the fact that no significant difference on mRNA levels of thiol biosynthetic enzymes was observed, increased levels of cysteine and glutathione for LgSbR and a higher amount of trypanothione in LbSbR were detected when compared to WT lines (Figure 2).

### Kinetics of Sb uptake and influx studies

**Figure 3:**
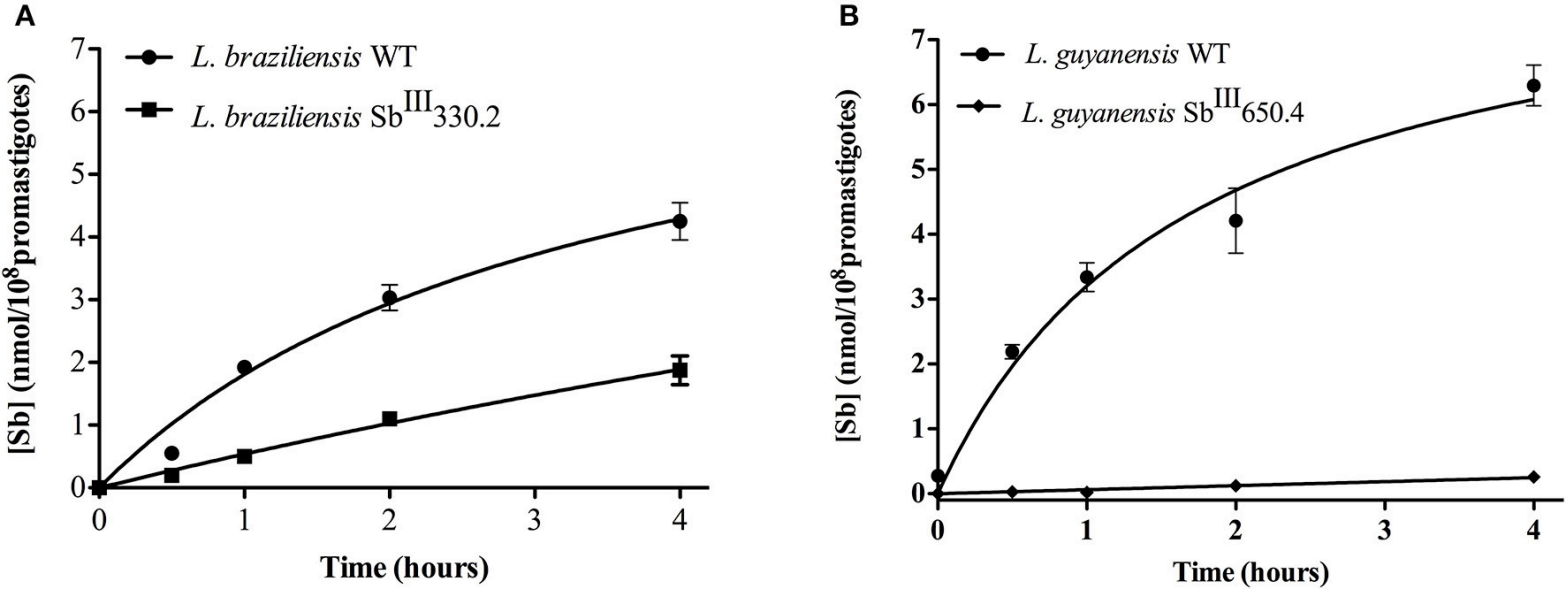
**Kinetics of incorporation of Sb^**III**^ uptake in WT and Sb^**III**^-resistant and ***L. braziliensis*** Sb^**III**^330.2 (A)** and *L. guyanensis* Sb^III^650.4 **(B)** promastigotes. Cells were incubated with 540 μM Sb^III^ at 25 ± 1°C under agitation shaking in Hepes/NaCl/Glucose buffer. Cells were harvested and washed at different time points for intracellular Sb determination by graphite furnace atomic absorption spectrometry. The values of Sb content are shown as means ± SEM (*n* = 8).

**Figure 4:**
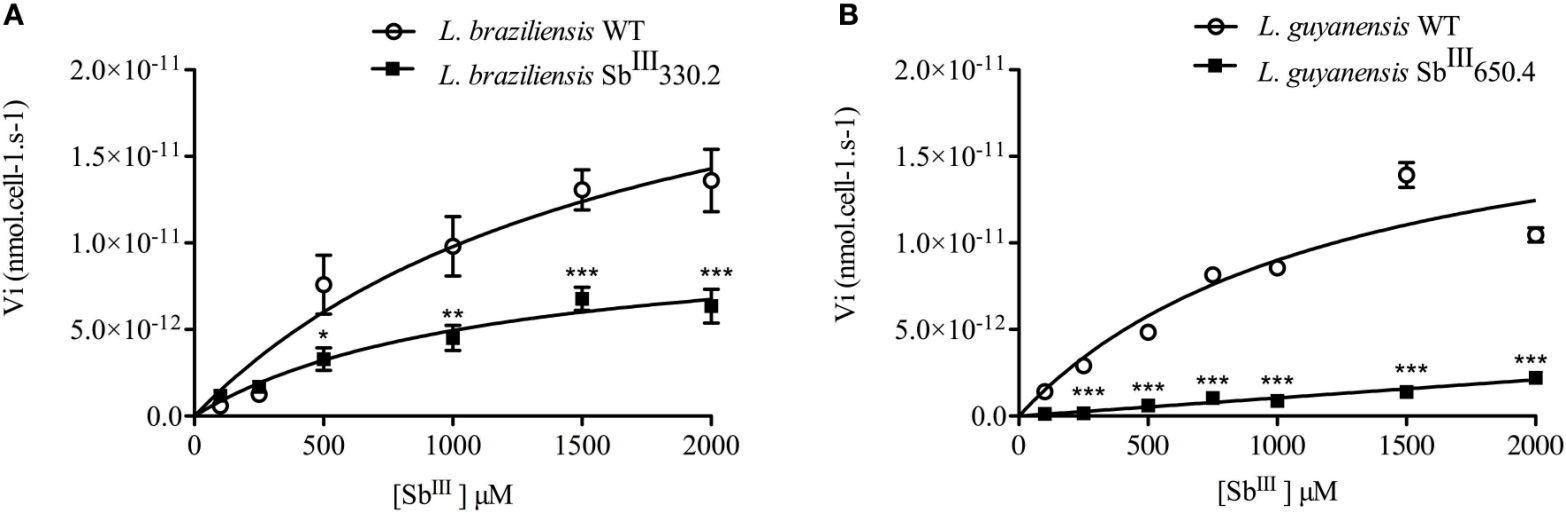
**Rate of Sb influx as a function of extracellular Sb concentration in WT and Sb^**III**^-resistant ***L. braziliensis*** Sb^**III**^330.2 (A)** and *L. guyanensis* Sb^III^650.4 **(B)**. The parasites were exposed to different concentrations of Sb^III^ for 1 h at 25°C in Hepes/NaCl/Glucose buffer and the intracellular Sb content was determined by graphite furnace atomic absorption spectroscopy. The influx rate of Sb was calculated as: Vi = amount of intracellular antimony/(number of cells x time of uptake). All experiments were performed at least three times as independent experiments in quadruplicate. The values are shown as means ± SEM. Statistically different values are highlighted as ^*^p < 0.05, ^**^p < 0.01, ^***^p < 0.001.

Figure 3 shows the kinetics of Sb uptake in *L. braziliensis* and *L. guyanensis* SbR mutants in comparison to their parental strains. The results indicate that both mutants exhibited a lower initial rate of Sb influx (3 × 10^−12^ and 0.4 × 10^−12^ nmol.s^−1^.cell^−1^ for *L. braziliensis* and *L. guyanensis*, respectively) when compared to their respective susceptible counterparts (7 × 10^−12^ and 5 × 10^−12^ nmol.s^−1^.cell^−1^, for *L. braziliensis* and *L. guyanensis*, respectively). As illustrated in Figure 4, the determination of the initial rate of Sb influx as a function of the extracellular Sb concentration showed a saturation at high drug concentration in the case of all strains, except for LgSbR. The kinetics constants of Sb influx were then calculated according to the Michaelis-Menten model (Table 3). We can infer that the decrease of drug influx contributed to the reduction of drug uptake, mainly in the case of LgSbR.

**Table 3:** **Kinetic constants for Sb influx at 25°C in WT and Sb^**III**^-resistant ***L. braziliensis*** and ***L. guyanensis*** promastigotes**.

***Leishmania* lines**	**INFLUX**			
	**^a^K_m_ (nM)**	**^a^V_max_ (nmol. s^−1^.cell^−1^)**	**^b^k_influx_ (L.s^−1^.cell^−1^)**	**k_influx_ WT/k_influx_R**
***L. braziliensis***				
Wild-type	1.7 ± 1.0 × 10^6^	2.6 ± 0.2 × 10^−11^	1.6 × 10^−17^	1.8
Resistant	1.1 ± 0.6 × 10^6^	1.1 ± 0.1 × 10^−11^	0.9 × 10^−17^	
***L. guyanensis***				
Wild-type	1.3 ± 0.3 × 10^6^	2.0 ± 0.1 × 10^−11^	1.6 × 10^−17^	166
Resistant	−	−	0.1 × 10^−18^	

a *The values were obtained by nonlinear regression analysis according to the Michaelis-Menten model*.

b *The values were calculated through k_influx_ = V_max_/K_m_ (Michaelis-Menten model) or through k_influx_ = Vi/Ce when no saturation was observed*.

### Kinetics of Sb efflux

**Figure 5:**
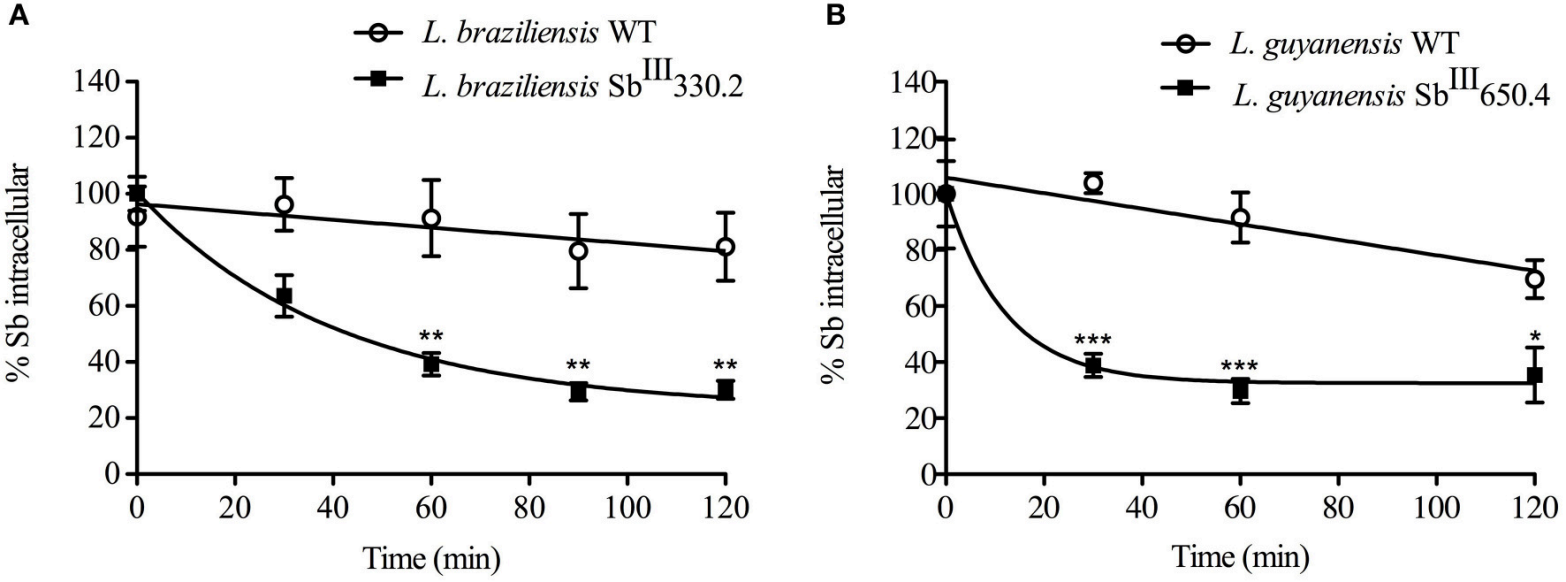
**Time course of Sb efflux at 25°C from WT and Sb^**III**^-resistant ***L. braziliensis*** Sb^**III**^330.2 (A)** and *L. guyanensis* Sb^III^650.4 **(B)** promastigotes. Cells were first loaded for 1 h with Sb^III^ and then washed, resuspended and incubated in drug-free Hepes/NaCl/Glucose buffer. After different time intervals (from 0 to 2 h), cells were retrieved and the amount of intracellular Sb was determined by graphite furnace atomic absorption spectroscopy. Data are expressed as percentage of initial Sb and shown as means ± SEM. All experiments were carried out at least three times as independent experiments performed in quadruplicate. Statistically different values are highlighted as ^*^p < 0.05, ^**^p < 0.01, ^***^p < 0.001.

After loading the mutant and WT promastigotes with a similar amount of Sb (the initial amounts of Sb were respectively: 0.50 ± 0.018 nmol/10^8^ cells and 0.53 ± 0.021 nmol/10^8^ cells for *L. guyanensis* WT and mutant; and 2.37 ± 0.53 nmol/10^8^ cells and 2.28 ± 0.61 nmol/10^8^ cells for *L. braziliensis* WT and mutant), the cells were washed and resuspended in a drug-free buffer to assess the kinetic of drug release. As shown in Figure 5, the Sb efflux was faster from the SbR mutants when compared with their WT counterparts. Assuming a monoexponential drug release model, the half-time of drug release was estimated and compared between the different cell lines (Table 4). The efflux of Sb was found 45-fold and 21-fold faster in LgSbR and LbSbR, respectively. These data support the model in which the increase of drug efflux strongly contributes to the reduction of drug uptake in both *Leishmania* mutants.

**Table 4:** **Kinetic constants for Sb^**III**^ efflux at 25°C in WT and Sb^**III**^-resistant ***L. braziliensis*** and ***L. guyanensis*** promastigotes**.

***Leishmania* lines**	**EFFLUX**		
	**^a^k_efflux_ (L.s^−1^.cell^−1^)**	**^b^T_1/2_ (s)**	**T_1/2_Wt/ T_1/2_R**
***L. braziliensis***			
Wild-type	1.4 × 10^−17^	593.5	21.0
Resistant	8.6 × 10^−17^	28.3	
***L. guyanensis***			
Wild-type	1.0 × 10^−17^	379.8	45.0
Resistant	13.7 × 10^−17^	8.4	

a *The efflux rate constant k_efflux_ was obtained from the equation: Ve = k_efflux_ × Ci, where Ve is the initial rate of efflux determined from the tangent of the kinetic curve and Ci is the intracellular concentration of Sb estimated at time zero using a cell volume of 1.2 × 10^−14^ L*.

b *The half-time of Sb efflux (T_1/2_) was calculated using mono-exponential decay model*.

### Effects of the ABC transporter inhibitors on Sb uptake and efflux in *Leishmania* spp.

To further characterize the Sb^III^ transport routes involved in the resistance of both *L. braziliensis* and *L. guyanensis Sb- resistant* mutants, a pharmacological approach was adopted based on the use of the following classical ABC transporter inhibitors: verapamil as MDR1-transporter inhibitor (Neal et al., 1989; Valiathan et al., 2006); probenicid as MRP-transporter inhibitor (Courtois et al., 1999; Payen et al., 2000; Mandal et al., 2009); prochlorperazine as MDR1- and MRP-transporter modulator (Essodaigui et al., 1999; Wesołowska, 2011; Rai et al., 2013) and BSO as intracellular thiol-depleting agent (Arana et al., 1998).

Table 5 displays the sensitivity (IC_50_) of the *L. braziliensis* and *L. guyanensis* strains to the different ABC transporter inhibitors (See also growth inhibition curves in Supplementary Figure 3). Although most of the inhibitors exhibited IC_50_ values in the same range when comparing the WT and SbR strains, prochlorperazine showed a distinct profile, as it was about 20-fold more active against the WT than LgSbR. This apparent cross-resistance suggests that prochlorperazine and Sb^III^ may share the same transport pathway in this mutant. Verapamil was also 2-fold more active against the WT than LgSbR, however, it showed an opposite profile in the *L. braziliensis* strains.

**Table 5:** **Sensitivity (IC_**50**_ ± SEM) of ***L. braziliensis*** and ***L. guyanensis*** strains to different ABC transporter inhibitors (verapamil, probenicid, prochlorperazine, BSO)**.

**Transporter**	**IC**_**50**_ ±**SEM**			
**inhibitors**	***L. braziliensis***		***L. guyanensis***	
	**Wild-type**	**Resistant**	**Wild-type**	**Resistant**
Verapamil (μM)	63.8 ± 1.8	27.0 ± 0.1	45.6 ± 3.4	82.7 ± 0.1
Prochlorperazine (μM)	7.5 ± 2.3	11.6 ± 0.1	3.5 ± 0.6	68.4 ± 0.1
Probenecid (mM)	204.5 ± 1.2	101.0 ± 0.2	503.5 ± 3.3	385.3 ± 31.5
BSO (mM)	>100	>100	>100	53.4 ± 0.8

The antileishmanial activities of Sb^III^ in the absence and presence of ABC transporter inhibitors were compared, for each tested *Leishmania* strain (Table 6 See also growth inhibition curves in Supplementary Figures 4,5). Among the different inhibitors, prochlorperazine was the only agent to resensitize both SbR strains, the most pronounced effect being observed in *L. guyanensis*. Interestingly, no such sensitization was observed in the WT parental strains. However, the lower concentration of prochlorperazine used in the WT lines (because of their greater susceptibility) may explain the lack of sensitization. Probenecid specifically sensitized the *L. braziliensis* strains to Sb^III^, but this effect occurred in both the mutant and wild-type strains. Surprisingly, verapamil promoted sensitization to Sb^III^ specifically in the WT strains.

**Table 6:** **Effect of different ABC transporter inhibitors^**a**^ on the half-maximal growth inhibition concentration (IC_**50**_) of Sb^III^ in Sb-resistant ***L. braziliensis*** and ***L. guyanensis*** promastigotes and their respective parental lines**.

**Transporter inhibitors**	**IC50 of SbIII ±SEM (μM)**			
	***L. braziliensis***		***L. guyanensis***	
	**Wild-type**	**Resistant**	**Wild-type**	**Resistant**
Sb^III^	86.1 ± 1.1	623.7 ± 44.7	47.4 ± 7.8	1167 ± 1.1
Verapamil	23.5 ± 3.5^**^	402.7 ± 18.2	9.5 ± 0.3^**^	877.7 ± 1.2
Prochlorperazine	78.0 ± 3.7	332.2 ± 17.4^*^	68.6 ± 1.4	146.4 ± 1.3^***^
Probenecid	45.7 ± 2.3^*^	330.0 ± 45.5^*^	51.9 ± 1.2	1163.4 ± 1.2
BSO	75.5 ± 3.0	567.8 ± 4.6	43.0 ± 4.5	945.3 ± 5.7

a *Non-toxic concentrations were used: 8 μM verapamil, 4 mM probenecid, 10 μM prochlorperazine, 100 μM BSO in resistant strains and 8 μM verapamil, 4 mM probenecid, 3.5 μM prochlorperazine, 100 μM BSO in parental strains*.

* *p < 0.05*,

** *p < 0.01*,

*** *p < 0.001 for statistical comparison to treatment with Sb^III^ alone. The data comes from at least three independent experiments*.

**Figure 6:**
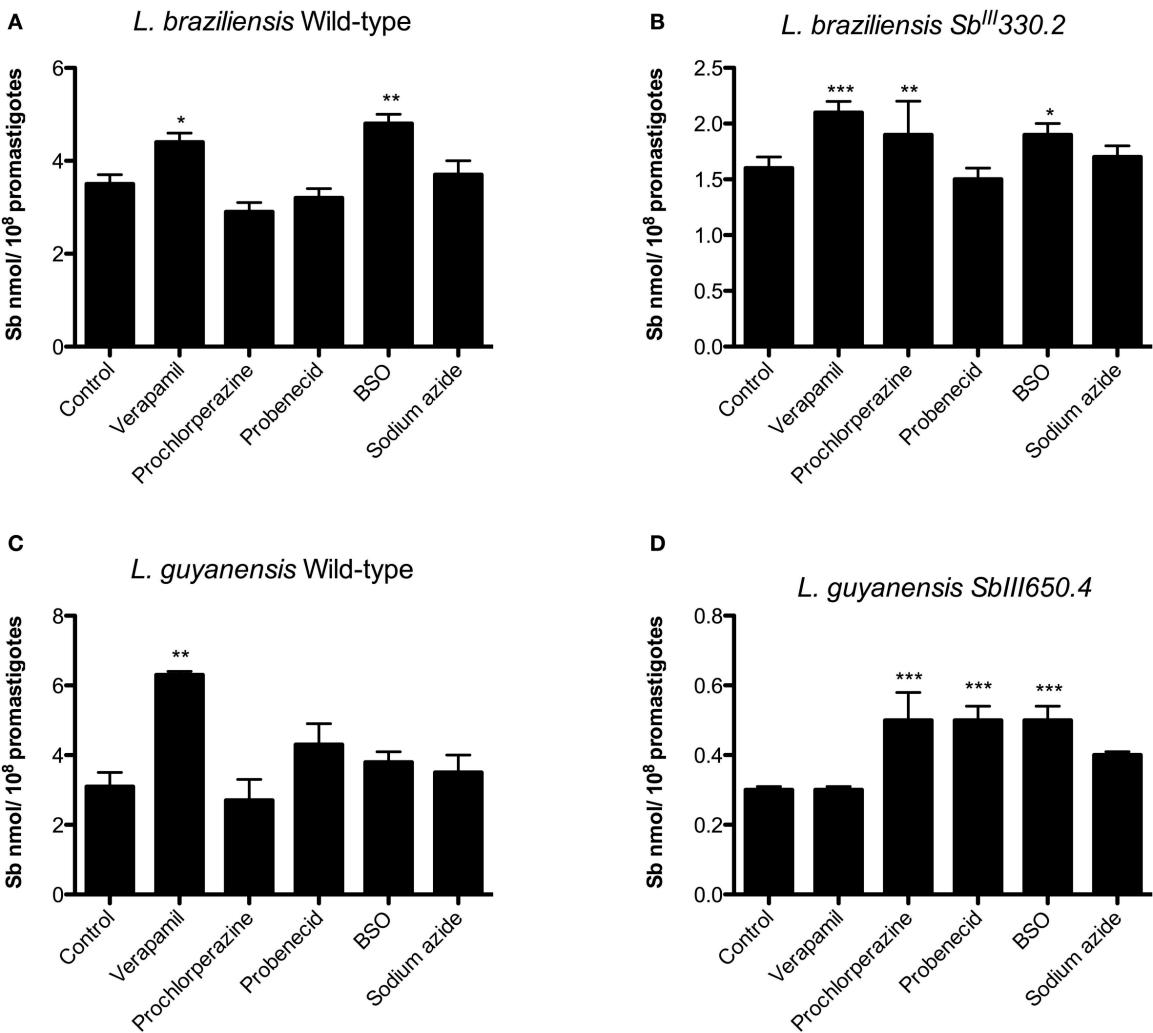
**Effect of pre-exposure to different ABC transporter inhibitors on Sb uptake in WT (A)** and Sb^III^-resistant **(B)** *L. braziliensis* and WT **(C)** and Sb^III^-resistant **(D)** *L. guyanensis* promastigotes. The cells were pre-incubated for 24 h in α-MEM in the absence or presence of the inhibitor (8 μM verapamil, 4 mM probenecid, 10 μM prochlorperazine, 100 μM BSO in resistant strains and 8 μM verapamil, 4 mM probenecid, 3.5 μM prochlorperazine, 100 μM BSO in parental strains), then exposed to 1 mM Sb^III^ for 1 h in HG buffer, washed and processed to determine the intracellular Sb content. When evaluating sodium azide, the cells were resuspended in glucose-free HG buffer containing 10 mM azide and 1 mM Sb^III^ and incubated for 1 h. The data comes from at least three independent experiments and are shown as means ± SEM. ^*^p < 0.05, ^**^p < 0.01, ^***^p < 0.001 for statistical comparison to Control, using One-way ANOVA followed by Bonferroni multiple comparison test.

Figure 6 shows the impact of cell pre-exposure to ABC transporter inhibitors on the subsequent Sb uptake. Prochlorperazine was the only inhibitor to enhance Sb uptake specifically in SbR strains, in agreement with its sensitizing effect. On the other hand, probenecid enhanced the uptake of Sb only in LgSbR. It is noteworthy that BSO resulted in increased Sb uptake in LgSbR mutant and in both WT and LbSbR. In contrast, exposition to the metabolic inhibitor sodium azide did not promote significant change in Sb uptake in any of the tested cell lines. Also, corroborating the results of the sensitization assay, verapamil increased the Sb uptake in the WT strains of both species.

**Figure 7:**
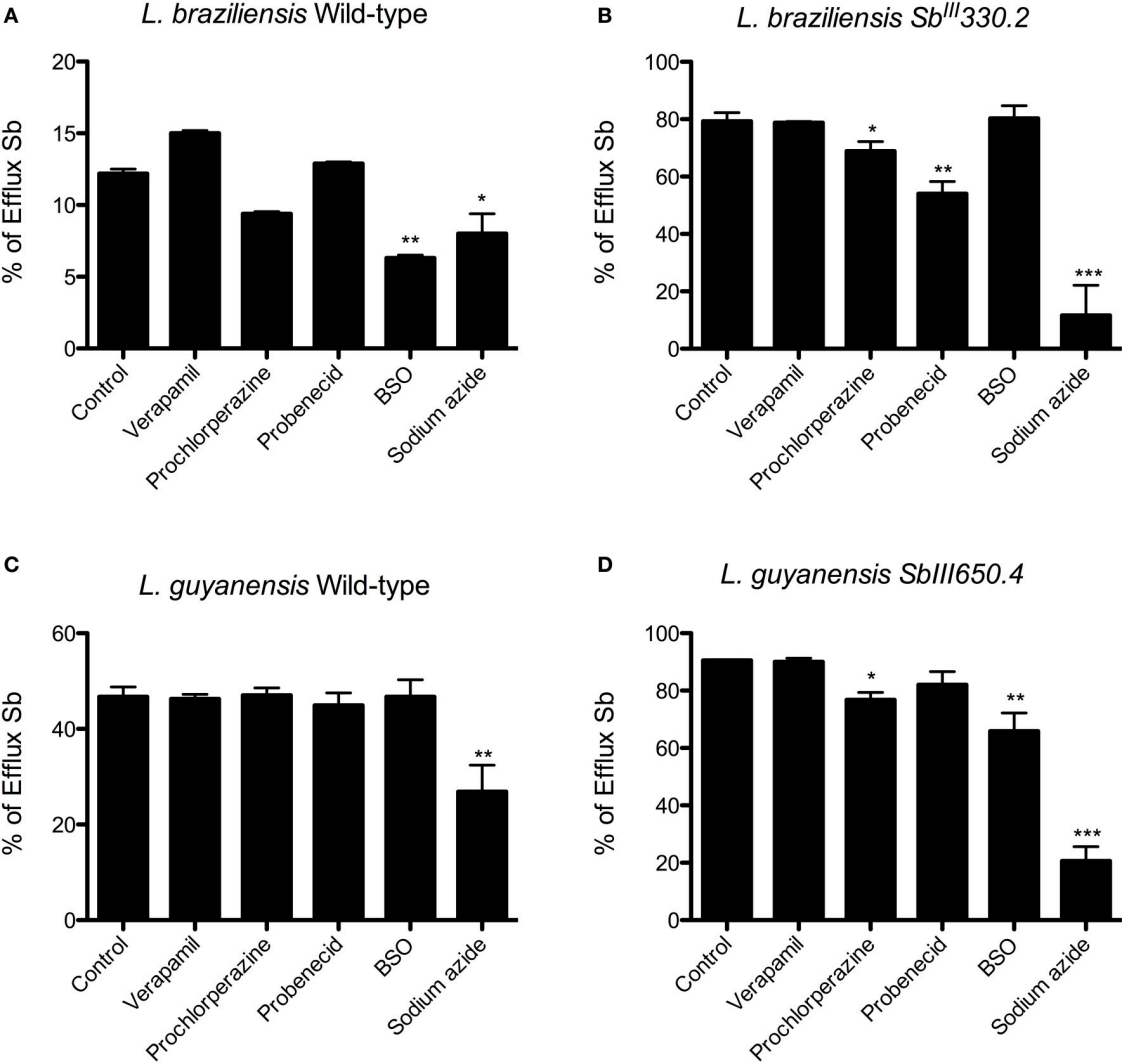
**Effect of different ABC transporter inhibitors on the percentage of Sb released from WT (A)** and Sb^III^-resistant **(B)** *L. braziliensis* and WT **(C)** and Sb^III^-resistant **(D)** *L. guyanensis* promastigotes, after 2 h of efflux. Cells were first exposed for 1 h at 25°C to 1 mM Sb^III^, were washed and resuspended in HG buffer at 108 cells/mL and were incubated for 2 h at 25°C in the absence or presence of the inhibitor (8 μM verapamil, 4 mM probenecid, 10 μM prochlorperazine, 100 μM BSO, 10 mM sodium azide in resistant strains and 8 μM verapamil, 4 mM probenecid, 3.5 μM prochlorperazine, 100 μM BSO, 10 mM sodium azide in parental strains). Only when evaluating the effect of azide, that the buffer did not contain glucose. The cells were finally washed and processed to determine the cellular content of Sb. The data are shown as the percentage of Sb released for 2 h in relation to the initial cellulart Sb content. The data comes from at least three independent experiments and are shown as means ± SEM. ^*^p < 0.05, ^**^p < 0.01, ^***^p < 0.001 for statistical comparison to Control (without inhibitor), using One-way ANOVA followed by Bonferroni multiple comparison test.

To evaluate the effect of the inhibitors on the efflux of Sb, cells were first exposed for 1 h to 1 mM Sb^III^, washed, resuspended in drug-free Hepes/NaCl buffer and incubated for 2 h in the absence or presence of the inhibitor. Figure 7 displays the percentages of Sb released from the different strains after 2 h of efflux. Prochlorperazine was the only agent to significantly inhibit Sb efflux specifically in the resistant strains, in agreement with its ability to increase the cellular drug uptake. Probenecid reduced the efflux of Sb in both mutants, but the effect was only significant in LbSbR. Sodium azide markedly inhibited the efflux of Sb mainly in SbR mutants, evidencing that Sb efflux in the resistant strains is essentially energy-dependent. The thiol-depleting agent BSO showed a significant effect only in LgSbR. On the other hand, verapamil exerted no significant effect on the drug efflux.

## Discussion

The main objective of the present work was to functionally characterize the transport routes of Sb in *L. braziliensis* and *L. guyanensis* strains selected for Sb resistance, by using systematic transport kinetic analysis and investigating the effect of ABC transporter inhibitors on uptake and efflux of Sb. This work can be seen as a continuation of a previous study performed on resistant laboratory mutants from the same Leishmania (Viannia) species, that also exhibited decreased influx and increased efflux of Sb (Moreira et al., 2013). New insights here include data on additional resistance markers such as mRNA levels of GSH1, LABCI4 and ARM58 and thiol levels, the demonstration of the energy-dependence of the efflux and the identification of a new chemosensitizer. In addition, the mutants studied here are different strains that were obtained independently in another laboratory. Indeed, our *L. braziliensis* mutant does not overexpress MRPA gene, contrary to the strain studied previously (Moreira et al., 2013).

The *L. guyanensis* mutant investigated previously showed down-regulation of AQP1 protein (Moreira et al., 2013), whereas the present mutant exhibits a single point mutation G133D in AQP1 (Monte-Neto et al., 2015). Functional validation confirmed that G133D mutation by itself is the main alteration related to reduced antimony uptake comparable with levels observed in *L. guyanensis* lacking *AQP1* (Monte-Neto et al., 2015). Since AQP1 also plays an important role in osmoregulation (Figarella et al., 2007), when submitted to a hypoosmotic challenge, *L. guyanensis* SbR mutants increased volume and presented a delayed recovery time compared to their WT counterpart, a profile that was also comparable with AQP1 lacking *L. guyanensis*, confirming the highly destabilizing nature of G133D mutation for AQP1 (R. Monte-Neto and D. Pires, unpublished results). The increased mRNA levels of *MRPA* and the marked reduction of Sb influx, as reported here, are consistent the previously reported amplification of the *MRPA* gene and the mutated and inactivated form of AQP1 (Monte-Neto et al., 2015).

Interestingly, the *L. braziliensis* SbR mutant did not show significant change in the mRNA levels of either *MRPA*, LABCI4, or ARM58 (Figure 1), suggesting that these transport proteins are not involved in the resistance mechanism of this strain. The lack of important change in antimony uptake in LbSbR mutant is in agreement with the unchanged AQP1 mRNA levels (Figure 1) together with the fact that gene sequence is intact (Supplementary Figure 1). Examining clinical isolates of *L. braziliensis* from brazilian patients presenting different antimonial treatment outcomes, Torres et al. (2010) did not find any difference in the expression levels of antimony metabolism associated genes such as MRPA, AQP1, GSH1, GSH2, TRYR, and TDR1 (Torres et al., 2010). It is noteworthy to mention that the resistance index (RI) of 7-fold presented by LbSbR (Table 2) is comparable with clinical isolates (Pérez et al., 2016) in which mechanisms of resistance would differ from those obtained from other laboratory-selected mutants presenting higher resistance indexes, like LgSbR that is approximately 25 times more resistant to Sb than its WT counterpart (Table 2). Indeed, laboratory-selected *L. braziliensis* presenting antimony RI of 20-fold had increased MRPA-encoding mRNA levels (Moreira et al., 2013), confirming the multifactorial nature of Sb resistance, being the mechanisms dependent on the RI.

Although no significant change was observed in mRNA levels of *GSH1* and *ODC*, increased levels of the intracellular thiols cysteine and glutathione were found in LgSbR, while LbSbR presented higher contents of trypanothione, when compared with their WT counterparts (Figure 2). A similar LgSbR thiol profile including increased levels of cysteine and glutathione without trypanothione change was also observed in SbR *L. donovani* field isolates (Mukherjee et al., 2007) and could be explained by a positive feedback where alterations in two thiol biosynthetic steps enhanced the amount of reduced trypanothione that can be depleted by a Sb-dependent mechanisms like Sb-TSH conjugate efflux (Wyllie et al., 2004). However, LbSbR presented an opposite profile, having higher trypanothione level and equivalent amounts of cysteine and glutathione, when compared with WT (Figure 2). Romero et al. (2015) reported an increase in total intracellular thiol content of *L. braziliensis* upon overexpression of cysteine synthase and cystathionine-β-synthase in presence of oxidative and nitrosative stresses (Romero et al., 2015). Thus, overexpression of other thiol biosynthetic enzyme(s) probably contributes to antimony resistance phenotype in our mutant. The increased amount of total intracellular thiols in absence of *GSH1* mRNA alterations as reported here was previously reported in clinical isolates of SbR *L. donovani* (Rai et al., 2013), also supporting the fact that the involvement of thiol metabolism in laboratory-selected SbR *Leishmania* (*Viannia*) species is a feature shared with field isolates.

From the transport kinetic studies, it is clear that both influx and efflux pathways contributed to the reduced cellular accumulation of Sb and the drug resistance phenotype. However, the change in drug influx had a much greater contribution in the *L. guyanensis* than in the *L. braziliensis* mutant. The fact that influx was not saturable in LgSbR, in contrast to the other strains, suggests that Sb^III^ enters into this cell through an AQP1-independent non-saturable transport route. A strongly energy-dependent efflux was clearly evidenced in both mutants. In LgSbR, the marked effect of BSO on Sb efflux (Figure 7) and the influence of probenecid on Sb uptake (Figure 6) further support the involvement of a MRP-type transporter capable of extruding metal-thiol conjugates. This transporter may be functionally related to MRPA found to be overexpressed in this mutant. Interestingly, the increased levels of thiols and MRPA mRNA in LgSbR also correlates with its higher sensitivity to BSO compared to its WT counterpart, in agreement with the observation of Moreira et al. (2013) for *L. braziliensis*. Whether the efflux transport also involves an exocytosis or a secretion pathway, as previously hypothesized (Legaré et al., 2001; Manzano et al., 2013; Perea et al., 2016; Tejera Nevado et al., 2016), still need to be investigated. In LbSbR, the effect of probenecid on the Sb efflux is also consistent with a MRP-type transporter, however, no increase in gene expression of the potential transporters MRPA, LABCI4 and ARM58 was observed (Figure 1). The lack of effect of BSO (Figure 7) also suggests no apparent dependence of the efflux on thiol, even though trypanothione showed higher levels in this mutant.

Verapamil significantly enhanced the sensitivity to Sb^III^ in the WT cell lines, but not in the mutants. In the parental cells, it also increased the uptake of Sb. On the other hand, no effect on Sb efflux was observed. Since verapamil was reported to inhibit the ABC transporter PRP1 in *L. major*, which was also found to confer low level of resistance to Sb^III^ (Coelho et al., 2003), a possible participation of PRP1 in the transport of Sb^III^ in the wild-type cells can be suggested.

The ability of prochlorperazine to specifically sensitize the resistant strains to Sb^III^ and inhibit the active efflux of Sb is an important finding of the present work. Due to its higher cytotoxicity toward WT than SbR strains, prochlorperazine was tested at lower concentration in the sensitization and transport assays of the WT strains. This may also explain the lack of effect of this drug in the WT strains. Our data strongly supports the model that this compound sensitizes the mutants to Sb by inhibiting the efflux route of Sb. However, one cannot completely discard the possibility that the sensitizing effect of prochlorperazine may come from the combined toxic effects of prochlorperazine and Sb^III^. Nevertheless, the efflux data in the resistant mutants which were co-exposed to prochlorperazine and Sb^III^ for only 2 h, strongly support a direct effect of prochlorperazine on efflux pathway. The lack of combined toxic effects is also reinforced by the fact that the WT strains which were more sensitive to both Sb^III^ and prochlorperazine did not exhibit any sensitizing effects. Prochlorperazine belongs to the class of phenothiazine drugs which have been found to be effective inhibitors of MDR1-transporters in cancer cells (Wesołowska, 2011; Takács et al., 2015). In Leishmania, these compounds were also reported to inhibit the efflux pumps of fluorescent substrates of ABC transporters (Essodaigui et al., 1999; Rai et al., 2013). However, it is the first report of a phenothiazine derivative capable of overcoming the antimony resistance in *Leishmania* parasites by interfering in the drug efflux. Regarding the mechanism of action of this compound, it may act either by competing with Sb^III^ for efflux transport or by reducing the intracellular concentration of reduced thiol through inhibition of the trypanothione reductase (Chan et al., 1998). The fact that prochlorperazine and Sb^III^ showed cross-resistance in the *L. guyanensis* mutant (Table 5) supports the idea that these drugs may share the same transport pathway and is consistent with the competition transport model.

In conclusion, the present study allowed the biophysical and pharmacological characterization of energy-dependent Sb efflux pathway apparently independent of *MRPA, ABCI4*, and *ARM58* upregulation, in a *Leishmania* (*Vianna*) mutant selected *in vitro* for resistance to Sb^III^. Prochlorperazine has also been identified as an effective chemosensitizer in both Sb resistant mutants, which acts through inhibition of the active efflux of Sb.

## Author contributions

PR performed the influx and efflux experiments and biological assays e wrote the first draft of the manuscript. RM selected the *L. braziliensis* and *L. guyanensis* mutants, performed the uptake experiment and qRT-PCR analysis and revised the manuscript. MM designed the biological assays and revised the manuscript. FF designed the whole study with emphasis on the transport experiments and wrote the final version of the manuscript.

## Funding

This work was supported by the Brazilian agencies Coordenação de Aperfeiçoamento de Pessoal de Nível Superior (2447/09), Conselho Nacional de Desenvolvimento Científico e Tecnológico (303227/2013-3), Fundação de Amparo à Pesquisa do Estado de Minas Gerais (CBB - APQ-01542-13 and RED-00007-14).

### Conflict of interest statement

The authors declare that the research was conducted in the absence of any commercial or financial relationships that could be construed as a potential conflict of interest.

## References

[B1] Alvar J. Velez I. D. Bern C. Herrero M. Desjeux P. Cano J.. (2012). Leishmaniasis worldwide and global estimates of its incidence. PLoS ONE 7:e35671. 10.1371/journal.pone.0035671 22693548 PMC3365071

[B2] Arana F. E. Pérez-Victoria J. M. Repetto Y. Morello A. Castanys S. Gamarro F. (1998). Involvement of thiol metabolism in resistance to glucantime in *Leishmania tropica*. Biochem. Pharmacol. 56, 1201–1208. 10.1016/S0006-2952(98)00129-4 9802332

[B3] Berg M. Vanaerschot M. Jankevics A. Cuypers B. Maes I. Mukherjee S.. (2013). Metabolic adaptations of *Leishmania donovani* in relation to differentiation, drug resistance, and drug pressure. Mol. Microbiol. 90, 428–442. 10.1111/mmi.12374 24020363

[B4] Bradford M. (1976). A rapid and sensitive method for the quantification of microgram quantities of protein utilizing the principle of protein-dye binding assay. Anal. Biochem. 72, 248–254. 10.1016/0003-2697(76)90527-3 942051

[B5] Brochu C. Wang J. Roy G. Messier N. Wang X. Y. Saravia N. G.. (2003). Antimony uptake systems in the protozoan parasite leishmania and accumulation differences in antimony-resistant parasites. Antimicrob. Agents Chemother. 47, 3073–3079. 10.1128/AAC.47.10.3073-3079.2003 14506011 PMC201146

[B6] Callahan H. L. Roberts W. L. Rainey P. M. Beverley S. M. (1994). The PGPA gene of *Leishmania major* mediates antimony [Sb^III^] resistance by decreasing influx and not by increasing efflux. Mol. Biochem. Parasitol. 68, 145–149. 10.1016/0166-6851(94)00154-5 7891738

[B7] Chan C. Yin H. Garforth J. McKie J. H. Jaouhari R. Speers P.. (1998). Phenothiazine inhibitors of trypanothione reductase as potential antitrypanosomal and antileishmanial drugs. J. Med. Chem. 41, 148–156. 10.1021/jm960814j 9457238

[B8] Cheng T. F. Sun H. Z. (2014). Chap 25. Antimony and bismuth, in Binding, Transport, and Storage of Metal Ions in Biological Cells, eds Wedd A. G. Maret W. (London, UK: The Royal Society of Chemistry), 768–799.

[B9] Coelho A. C. Beverley S. M. Cotrim P. C. (2003). Functional genetic identification of PRP1, an ABC transporter superfamily member conferring pentamidine resistance in *Leishmania major*. Mol. Biochem. 30, 83–90. 10.1016/s0166-6851(03)00162-2 12946844

[B10] Courtois A. Payen L. Lagadic D. Guillouzo A. Fardel O. (1999). Evidence for a multidrug resistance-associated protein 1 (MRP1)-related transport system in cultured rat liver biliary epithelial cells. Life Sci. 64, 763–774. 10.1016/S0024-3205(98)00618-3 10075109

[B11] Croft S. L. Sundar S. Fairlamb A. H. (2006). Drug resistance in leishmaniasis. Clin. Microbiol. Rev. 19, 111–126. 10.1128/CMR.19.1.111-126.2006 16418526 PMC1360270

[B12] Decuypere S. Rijal S. Yardley V. De Doncker S. Laurent T. Khanal B.. (2005). Gene expression analysis of the mechanism of natural Sb(V) resistance in *Leishmania donovani* isolates from Nepal. Antimicrob. Agents Chemother. 49, 4616–4621. 10.1128/AAC.49.11.4616-4621.2005 16251303 PMC1280167

[B13] Decuypere S. Vanaerschot M. Brunker K. Imamura H. Müller S. Khanal B.. (2012). Molecular mechanisms of drug resistance in natural *Leishmania* populations vary with genetic background. PLoS Negl. Trop. Dis. 6:e1514. 10.1371/journal.pntd.0001514 22389733 PMC3289598

[B14] Dey S. Papadopoulou B. Haimeur A. Roy G. Grondin K. Dou D.. (1994). High level arsenite resistance in *Leishmania tarentolae* is mediated by an active extrusion system. Mol. Biochem. Parasitol. 67, 49–57. 10.1016/0166-6851(94)90095-7 7838183

[B15] Do Monte-Neto R. L. Coelho A. C. Raymond F. Légaré D. Corbeil J. Melo M. N.. (2011). Gene expression profiling and molecular characterization of antimony resistance in *Leishmania amazonensis*. PLoS Negl. Trop. Dis. 5:e1167. 10.1371/journal.pntd.0001167 21629719 PMC3101167

[B16] Essodaigui M. Frézard F. Moreira E. S. A. Dagger F. Garnier-Suillerot A. (1999). Energy-dependent efflux from *Leishmania* promastigotes of substrates of the mammalian multidrug resistance pumps. Mol. Biochem. Parasitol. 100, 73–84. 10.1016/S0166-6851(99)00036-5 10376995

[B17] Fairlamb A. H. Henderson G. B. Bacchi C. J. Cerami A. (1987). *In vivo* effects of difluoromethylornithine on trypanothione and polyamine levels in bloodstream forms of Trypanosoma brucei. Mol. Biochem. Parasitol. 24, 185–191. 10.1016/0166-6851(87)90105-8 3114634

[B18] Figarella K. Uzcategui N. L. Zhou Y. LeFurgey A. Ouellette M. Bhattacharjee H.. (2007). Biochemical characterization of Leishmania major aquaglyceroporin LmAQP1: possible role in volume regulation and osmotaxis. Mol. Microbiol. 65, 1006–1017. 10.1111/j.1365-2958.2007.05845.x 17640270

[B19] Freshney R. I. (1994). Culture of Animal Cells: A Manual of Basic Technique. New York, NY: Wiley-Liss Publishers.

[B20] Frézard F. Demicheli C. Ribeiro R. R. (2009). Pentavalent antimonials: new perspectives for old drugs. Molecules 14, 2317–2336. 10.3390/molecules14072317 19633606 PMC6254722

[B21] Frézard F. Monte-Neto R. Reis P. G. (2014). Antimony transport mechanisms in resistant leishmania parasites. Biophys. Rev. 6, 119–132. 10.1007/s12551-013-0134-y PMC5425714 28509965

[B22] Fumarola L. Spinelli R. Brandonisio O. (2004). *In vitro* assays for evaluation of drug activity against Leishmania spp. Res. Microbiol. 155, 224–230. 10.1016/j.resmic.2004.01.001 15142618

[B23] Gazanion É. Fernández-Prada C. Papadopoulou B. Leprohon P. Ouellette M. (2016). Cos-Seq for high-throughput identification of drug target and resistance mechanisms in the protozoan parasite Leishmania. Proc. Natl. Acad. Sci. U.S.A. 113, E3012–E3021. 10.1073/pnas.1520693113 27162331 PMC4889358

[B24] Hadighi R. Mohebali M. Boucher P. Hajjaran H. Khamesipour A. Ouellette M. (2006). Unresponsiveness to Glucantime treatment in Iranian cutaneous leishmaniasis due to drug-resistant *Leishmania tropica* parasites. PLoS Med. 3:e162. 10.1371/journal.pmed.0030162 16605301 PMC1435779

[B25] Kazemi-Rad E. Mohebali M. Khadem-Erfan M. B. Saffari M. Raoofian R. Hajjaran H.. (2013). Identification of antimony resistance markers in *Leishmania tropica* field isolates through a cDNA-AFLP approach. Exp. Parasitol. 135, 344–349. 10.1016/j.exppara.2013.07.018 23928349

[B26] Kumar D. Singh R. Bhandari V. Kulshrestha A. Negi N. S. Salotra P. (2012). Biomarkers of antimony resistance: need for expression analysis of multiple genes to distinguish resistance phenotype in clinical isolates of *Leishmania donovani*. Parasitol. Res. 111, 223–230. 10.1007/s00436-012-2823-z 22302478

[B27] Legaré D. Richard D. Mukhopadhyay R. Stierhof Y. D. Rosen B. P. Haimeur A.. (2001). The Leishmania ATP-binding cassette protein PGPA is an intracellular metal-thiol transporter ATPase. J. Biol. Chem. 276, 26301–26307. 10.1074/jbc.M102351200 11306588

[B28] Lira R. Sundar S. Makharia A. Kenney R. Gam A. Saraiva E.. (1999). Evidence that incidence of treatment failure in Indian kala-azar is due to the emergence of antimony resistant strains of *Leishmania donovani*. J. Infect. Dis. 180, 564–567. 10.1086/314896 10395884

[B29] Mandal G. Sarkar A. Saha P. Singh N. Sundar S. Chartterjee M. (2009). Functionality of drug efflux pumps in antimonial resistant *Leishmania donovani* field isolates. Indian J. Biochem. Biophys. 46, 86–92. Available online at: http://nopr.niscair.res.in/handle/123456789/3322 19374259

[B30] Manzano J. I. Garcia-Hernandez R. Castanys S. Gamarro F. (2013). A new ABC half-transporter in Leishmania is involved in resistance to antimony. Antimicrob. Agents Chemother. 57, 3719–3730. 10.1128/AAC.00211-13 23716044 PMC3719772

[B31] Marquis N. Gourbal B. Rosen B. P. Mukhopadhyay R. Ouellette M. (2005). Modulation in aquaglyceroporin AQP1 gene transcript levels in drug-resistant Leishmania. Mol. Microbiol. 57, 1690–1699. 10.1111/j.1365-2958.2005.04782.x 16135234

[B32] Marzochi M. C. Marzochi K. B. (1994). Tegumentary and visceral leishmaniases in Brazil: emerging anthropozoonosis and possibilities for their control. Cad. Saude Publica 10, 2359–2375. 10.1590/s0102-311x1994000800014 15042226

[B33] Monte-Neto R. Laffitte M. C. Leprohon P. Reis P. G. Frézard F. Ouellette M. (2015). Intrachromosomal amplification, locus deletion and point mutation in the aquaglyceroporin AQP1 gene in antimony resistant Leishmania (Viannia) guyanensis. PLoS Negl. Trop. Dis. 9:e0003476. 10.1371/journal.pntd.0003476 25679388 PMC4332685

[B34] Moreira D. S. Monte-Neto R. L. Andrade J. M. Santi A. M. M. Reis P. G. Frézard F.. (2013). Molecular characterization of the MRPA transporter and antimony uptake in four New World Leishmania spp. susceptible and resistant to antimony. Int. J. Parasitol. Drugs Drug Resist. 3, 143–153. 10.1016/j.ijpddr.2013.08.001 24533304 PMC3862441

[B35] Mukherjee A. Padmanabhan P. K. Singh S. Roy G. Girard I. Chatterjee M.. (2007). Role of ABC transporter MRPA, gamma-glutamylcysteine synthetase and ornithine decarboxylase in natural antimony-resistant isolates of *Leishmania donovani*. J. Antimicrob. Chemother. 59, 204–211. 10.1093/jac/dkl494 17213267

[B36] Mukhopadhyay R. Dey S. Xu N. Gage D. Lightbody J. Ouellette M.. (1996). Trypanothione overproduction and resistance to antimonials and arsenicals in *Leishmania*. Proc. Natl. Acad. Sci. U.S.A. 93, 10383–10387. 10.1073/pnas.93.19.10383 8816809 PMC38393

[B37] Murray H. W. Berman J. D. Davies C. R. Saravia N. G. (2005). Advances in leishmaniasis. Lancet 366, 1561–1577. 10.1016/S0140-6736(05)67629-5 16257344

[B38] Neal R. A. Van Bueren J. McCoy N. G. Iwobi M. (1989). Reversal of drug resistance in Trypanosoma cruzi and *Leishmania donovani* by verapamil. Trans. R. Soc. Trop. Med. Hyg. 83, 197–198. 10.1016/0035-9203(89)90642-1 2558433

[B39] Nühs A. Schäfer C. Zander D. Trübe L. Tejera Nevado P. Schmidt S.. (2013). A novel marker, ARM58, confers antimony resistance to Leishmania spp. Int. J. Parasitol. Drugs Drug Resist. 4, 37–47. 10.1016/j.ijpddr.2013.11.004 24596667 PMC3940081

[B40] Ouellette M. Drummelsmith J. Papadopoulou B. (2004). Leishmaniasis: drugs in the clinic, resistance and new developments. Drug Resist. Updat. 7, 257–266. 10.1016/j.drup.2004.07.002 15533763

[B41] Papadopoulou B. Roy G. Dey S. Rosen B. P. Ouellette M. (1994). Contribution of the *Leishmania* P-glycoprotein-related gene ltpgpA to oxyanion resistance. J. Biol. Chem. 269, 11980–11986. 7909316

[B42] Payen L. Courtois A. Campion J. P. Guillouzo A. Fardel O. (2000). Characterization and inhibition by a wide range of xenobiotics of organic anion excretion by primary human hepatocytes. Biochem. Pharmacol. 60, 1967–1975. 10.1016/S0006-2952(00)00496-2 11108814

[B43] Perea A. Manzano J. I. Castanys S. Gamarro F. (2016). The LABCG2 transporter from the protozoan parasite *Leishmania* is involved in antimony resistance. Antimicrob. Agents Chemother. 60, 3489–3496. 10.1128/AAC.02813-15 27021316 PMC4879363

[B44] Pérez V. G. García-Hernandez R. Corpas-López V. Tomás A. M. Martín-Sanchez J. Castanys S.. (2016). Decreased antimony uptake and overexpression of genes of thiol metabolism are associated with drug resistance in a canine isolate of *Leishmania infantum*. Int. J. Parasitol. Drugs Drug Resist. 6, 133–139. 10.1016/j.ijpddr.2016.04.003 27317865 PMC4919363

[B45] Perry M. R. Wyllie S. Prajapati V. K. Feldmann J. Sundar S. Boelaert M.. (2011). Visceral leishmaniasis and arsenic: an ancient poison contributing to antimonial treatment failure in the Indian subcontinent? PLoS Negl. Trop. Dis. 5:e1227. 10.1371/journal.pntd.0001227 21980542 PMC3181240

[B46] Rai S. Bhaskar G. S. K. Nath Dwivedi U. Sundar S. Goyal N. (2013). Role of efflux pumps and intracellular thiols in natural antimony resistant isolates of *Leishmania donovani*. PLoS ONE 8:e74862. 10.1371/journal.pone.0074862 24069359 PMC3775726

[B47] Roberts W. L. Rainey P. M. (1993). Antileishmanial activity of sodium stibogluconate fractions. Antimicrob. Agents Chemother. 37, 1842–1846. 10.1128/AAC.37.9.1842 8239593 PMC188079

[B48] Romero I. Téllez J. Romanha A. J. Steindel M. Grisard E. C. (2015). Upregulation of cysteine and cystathionine-β-synthase contributes to *Leishmania brasiliensis* survival under oxidative stress. Antimicrob. Agents Chemother. 59, 4770–4781. 10.1128/AAC.04880-14 26033728 PMC4505290

[B49] Schäfer C. Tejera Nevado P. Zander D. Clos J. (2014). Reduced antimony accumulation in ARM58-overexpressing *Leishmania infantum*. Antimicrob. Agents Chemother. 58, 1565–1574. 10.1128/AAC.01881-13 24366738 PMC3957878

[B50] Takács D. Csonka Á. Horváth Á. Windt T. Gajdács M. Riedl Z.. (2015). Reversal of ABCB1-related multidrug resistance of colonic adenocarcinoma cells by phenothiazines. Anticancer Res. 35, 3245–3251. Available online at: http://ar.iiarjournals.org/content/35/6/3245.short 26026084

[B51] Tejera Nevado P. Bifeld E. Höhn K. Clos J. (2016). A telomeric cluster of antimony resistance genes on chromosome 34 of *Leishmania infantum*. Antimicrob. Agents Chemother. 60, 5262–5275. 10.1128/AAC.00544-16 27324767 PMC4997884

[B52] Torres D. C. Adaui V. Ribeiro-Alves M. Romero G. A. Arévalo J. Cupolillo E.. (2010). Targeted gene expression profiling in *Leishmania brasiliensis* and *Leishmania guyanensis* parasites isolated from brazilian patients with different antimonial treatment outcomes. Infect. Genet. Evol. 10, 727–733. 10.1016/j.meegid.2010.05.006 20478409

[B53] Valiathan R. Dubey M. L. Mahajan R. C. Malla N. (2006). *Leishmania donovani*: effect of verapamil on *in vitro* susceptibility of promastigote and amastigote stages of Indian clinical isolates to sodium stibogluconate. Exp. Parasitol. 114, 103–108. 10.1016/j.exppara.2006.02.015 16616137

[B54] Wesołowska O. (2011). Interaction of phenothiazines, stilbenes and flavonoids with multidrug resistance-associated transporters, P-glycoprotein and MRP1. Acta Biochim. Pol. 58, 433–448. Available online at: http://www.actabp.pl/pdf/4_2011/433.pdf 22187677

[B55] Wyllie S. Cunningham M. L. Fairlamb A. H. (2004). Dual action of antimonial drugs on thiol redox metabolism in human pathogen *Leishmania donovani*. J. Biol. Chem. 279, 39925–39932. 10.1074/jbc.M405635200 15252045

[B56] Zilberstein D. Dwyer D. M. (1984). Glucose transport in *Leishmania donovani* promastigotes. Mol. Biochem. Parasitol. 12, 327–336. 10.1016/0166-6851(84)90089-6 6482908

