# Supplementary material for: Biophysical and Pharmacological Characterization of Energy-Dependent Efflux of Sb in Laboratory-Selected Resistant Strains of Leishmania (Viannia) Subgenus
Source: Front Cell Dev Biol. 2017 Mar 24;5:24. doi: 10.3389/fcell.2017.00024 (PMC5364148; doi:10.3389/fcell.2017.00024)
Supplement: Supplementary file 1 [file Presentation1.PDF]

Supplementary Material

Biophysical and pharmacological characterization of energy-dependent efflux of Sb in laboratory-selected resistant strains of Leishmania (Viannia) subgenus

Priscila Gomes dos Reis, Rubens Lima do Monte-Neto, Maria Norma Melo, Frédéric Frézard*

*** Correspondence:** Corresponding Author: frezard@icb.ufmg.br

1

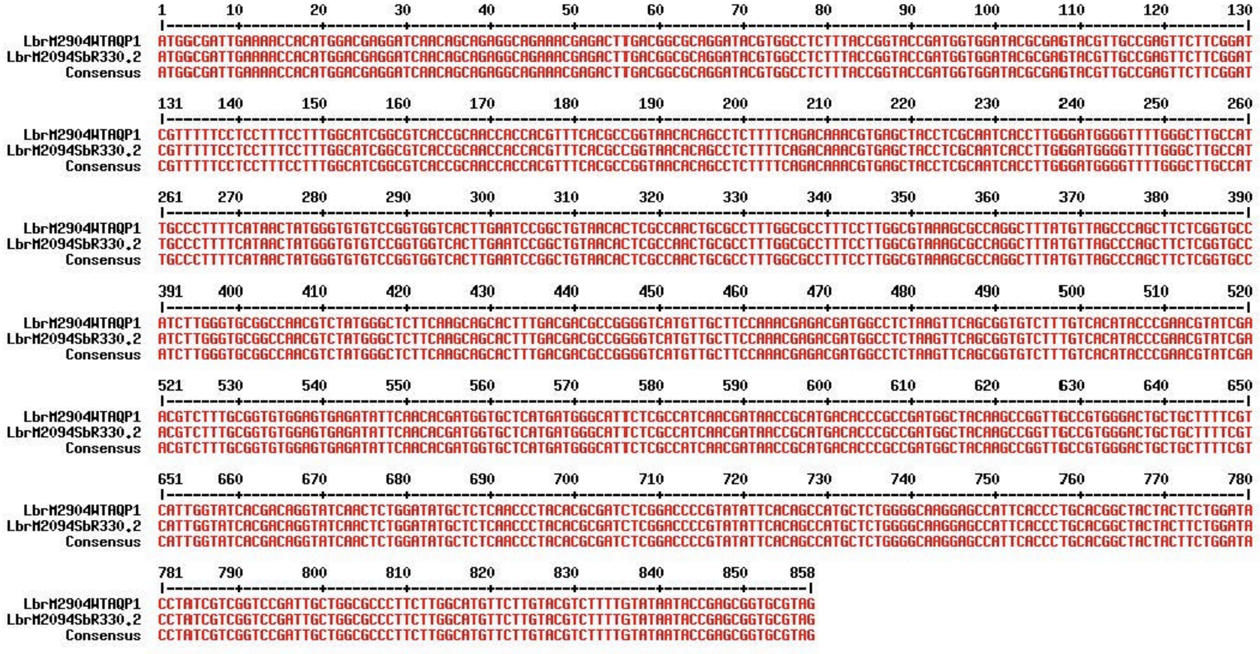

Supplementary Figure 1. Multiple alignment of AQP1 gene sequence from *L. braziliensis* WT (line 1) and *L. braziliensis* Sb^{III}330.2 (line 2). Powered by: http://multalin.toulouse.inra.fr/multalin/

2

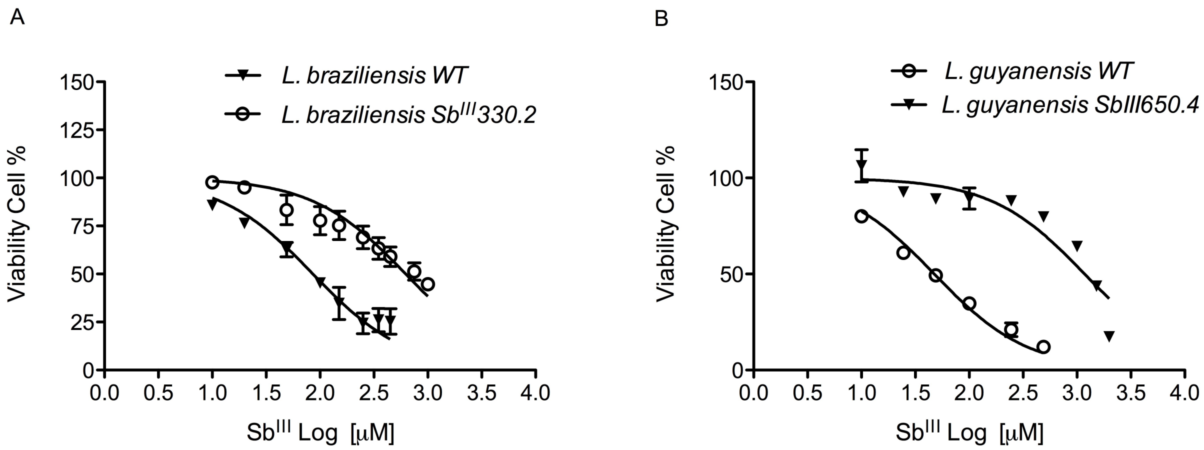

Supplementary Figure 2. Growth inhibition curves of Sb-resistant *L. braziliensis* (**A**) and *L. guyanensis* (**B**) promastigotes and their respective parental (WT) lines, after exposition for 72 h to increasing concentration of Sb^{III}. Data are shown as means ± SEM of three independent experiments.

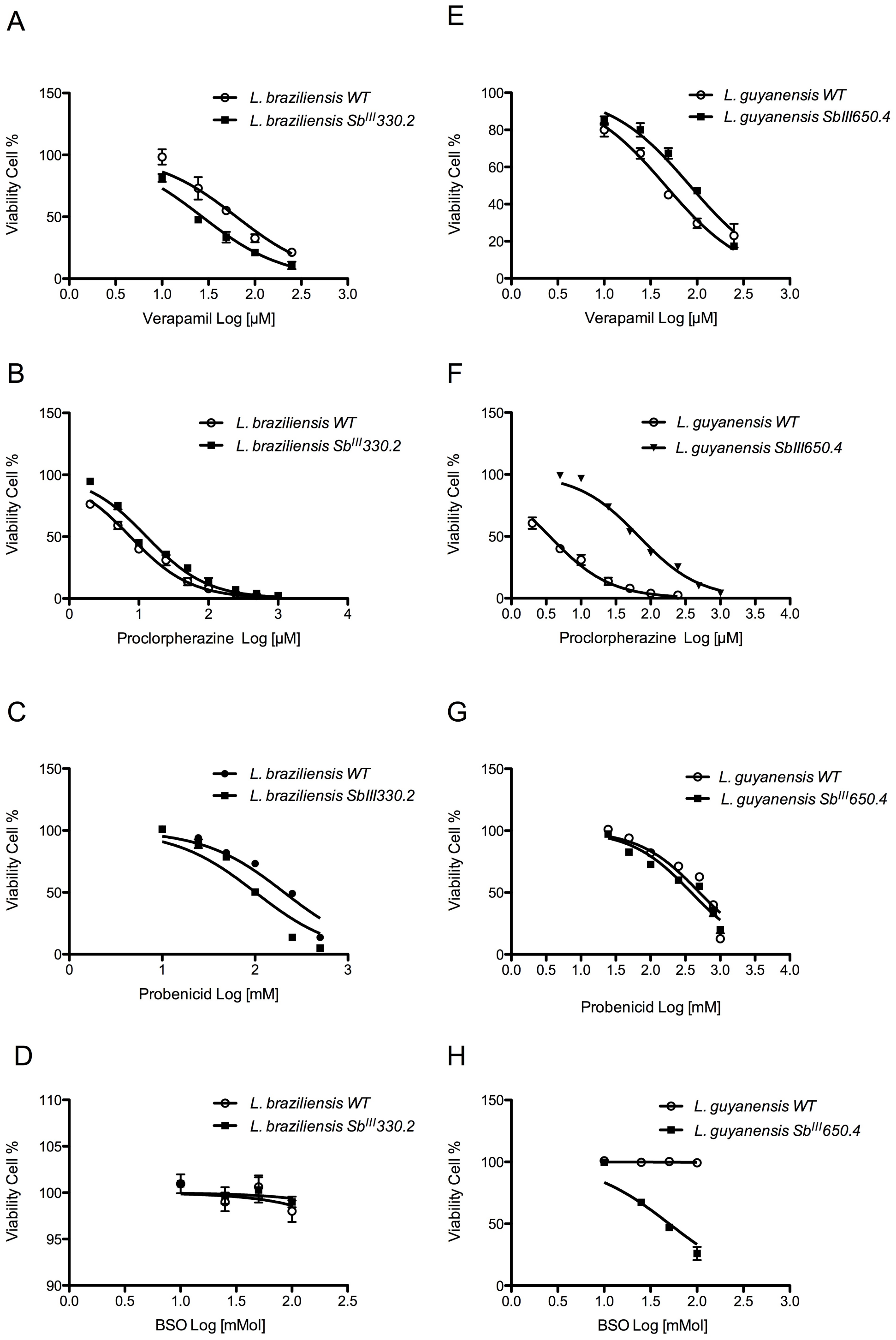

Supplementary Figure 3. Growth inhibition curves of Sb-resistant *L. braziliensis* and *L. guyanensis* promastigotes and their respective parental (WT) lines after exposition for 72 h to increasing concentrations of verapamil (**A, E**), prochlorperazine (**B, F**), probenecid (**C, G**) and BSO (**D, H**). Data are shown as means ± SEM of three independent experiments.

4

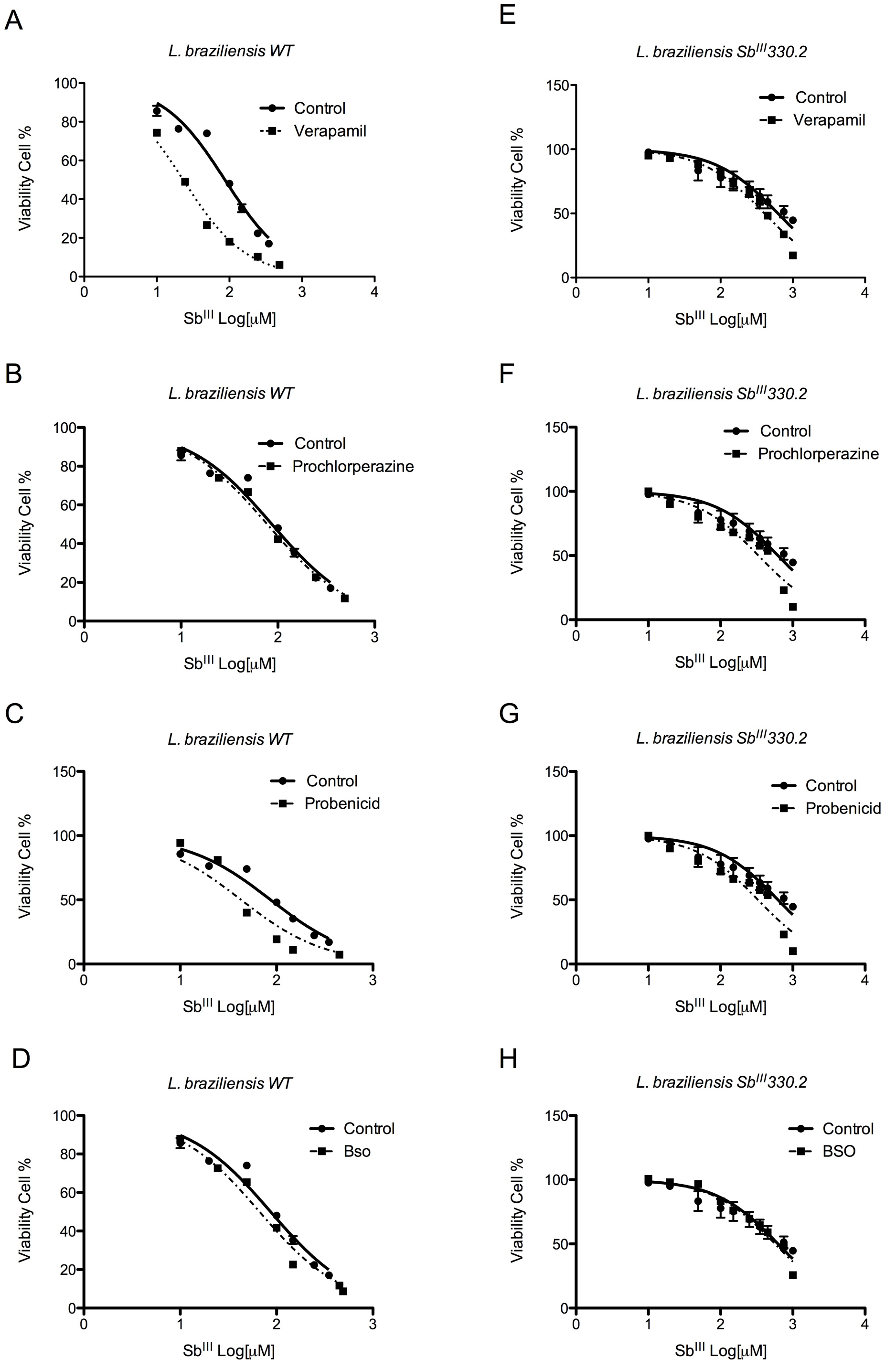

Supplementary Figure 4. Growth inhibition curves of Sb-resistant *L. braziliensis* and its respective parental (WT) line, after exposition for 72 h to increasing concentration of Sb^{III} in the absence (control) or presence of verapamil (**A, E**), prochlorperazine (**B, F**), probenecid (**C, G**) and BSO (**D, H**). Data are shown as means ± SEM of three independent experiments.

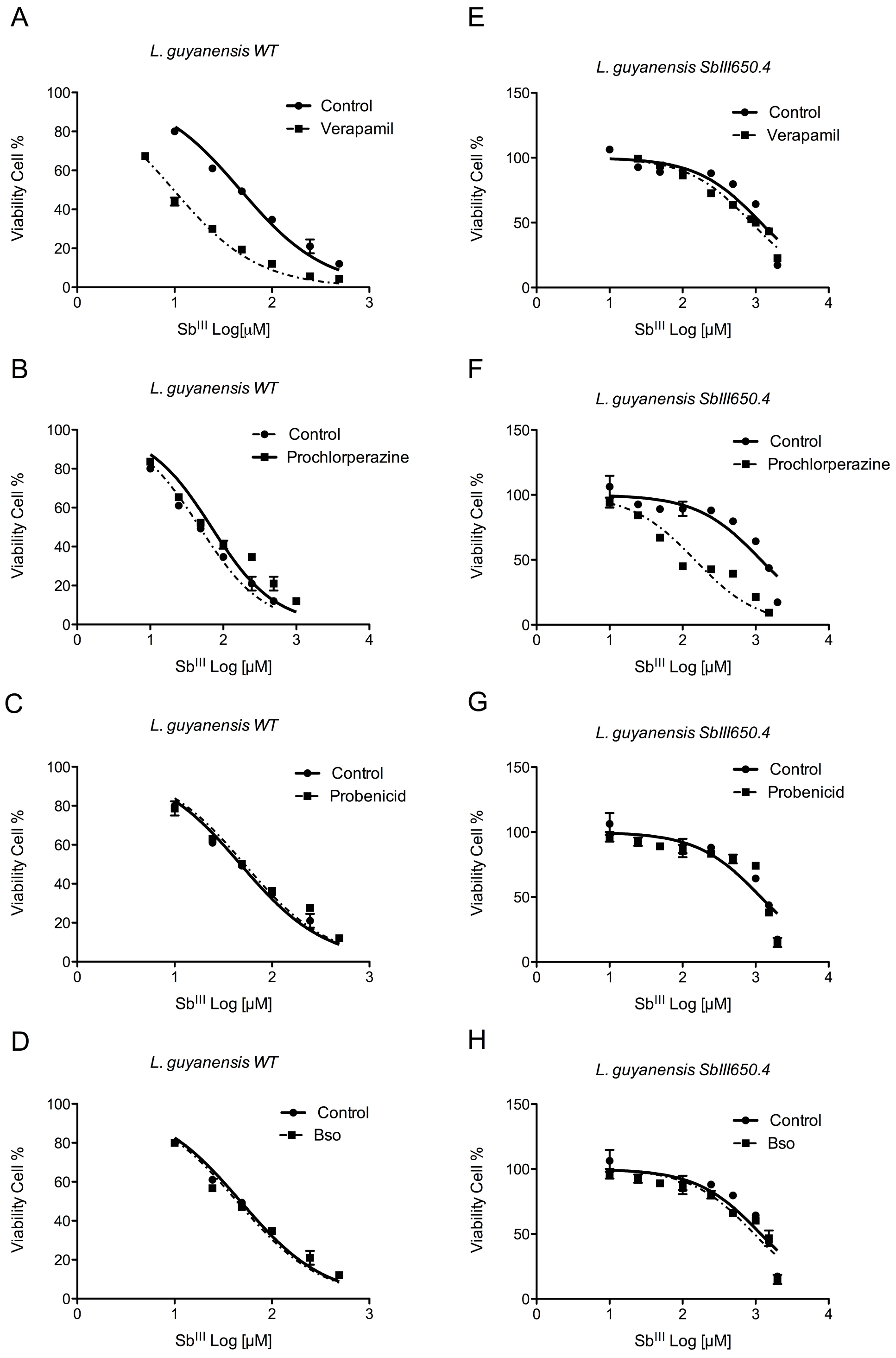

Supplementary Figure 5. Growth inhibition curves of Sb-resistant *L. guyanensis* and its respective parental (WT) line, after exposition for 72 h to increasing concentration of Sb^{III} in the absence (control) or presence of verapamil (**A, E**), prochlorperazine (**B, F**), probenecid (**C, G**) and BSO (**D, H**). Data are shown as means ± SEM of three independent experiments.
